# New isoflavonoids from *Erythrina arborescens* and structure revision of anagyroidisoflavone A

**DOI:** 10.1007/s13659-013-0062-3

**Published:** 2013-10-14

**Authors:** Fei Wang, Xu-Long Li, Guo-Zhu Wei, Fu-Cai Ren, Ji-Kai Liu

**Affiliations:** 1 BioBioPha Co., Ltd., Kunming, 650201 China; 2 State Key Laboratory of Phytochemistry and Plant Resources in West China, Kunming Institute of Botany, Chinese Academy of Sciences, Kunming, 650201 China

**Keywords:** *Erythrina arborescens*, isoflavonoid, erythrinin, structure revision

## Abstract

Five hitherto unknown isoflavonoids, namely erythrinins D-H (**1–5**), were isolated from the ethanol extract of *Erythrina arborescens*. Their structures were elucidated on the basis of extensive spectroscopic studies. In addition, the structure of anagyroidisoflavone A (**6a**) has been revised as 1″-*O*-methylerythrinin F (**6**) by re-analysis of the original spectroscopic data.

**Electronic Supplementary Material:**

Supplementary material is available for this article at 10.1007/s13659-013-0062-3 and is accessible for authorized users.

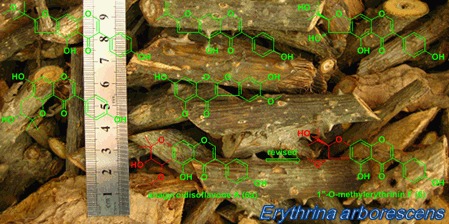

## Introduction

The genus *Erythrina* (Fabaceae) contains about 130 species, which are distributed in tropical and subtropical regions worldwide. The origin of the name *Erythrina* comes from the Greek word "erythros", meaning red, alluding to the bright red flowers of many species of the genus. The bark and leaves of *Erythrina* species are commonly utilized for a wide variety of human diseases in folk medicine, and many ethnopharmacological studies have been performed in order to confirm the anecdotal evidence attributed to these species.[1] Previous studies show the genus is a rich source of bioactive alkaloids and flavonoids, especially, isoflavones, pterocarpans and flavanones.[1,2] As part of a BioBioPha [http://www.biobiopha.com/Enindex.html] objective to assemble a large-scale natural product library valuable in the discovery of new drug leads from nature,[3] the phytochemical investigation of the non-alkaloidal components of the twigs of *Erythrina arborescens* led to isolation of five new isoflavonoids, namely erythrinins D–H (**1**–**5**). This paper describes the isolation and structural elucidation of these new compounds and structure revision of an isoflavone anagyroidisoflavone A (**6a**).

## Results and Discussion

Compound **1** was obtained as a yellow amorphous powder, and has molecular formula of $C_{21}H_{18}O_6$ based on HREIMS, showing a molecular ion peak at *m*/*z* 366.1099 (calcd for

**1**

**2**

**3**

**4**

**5**

$C_{21}H_{18}O_6$, 366.1103). The $^1$H NMR spectrum (Table 1) showed a set of signals at $\delta_H$ 7.41 and 6.83 (each 2H, d, $J$ = 8.5 Hz) ascribed to a *p*-substituted phenyl, three aromatic or olefinic proton singlets at $\delta_H$ 8.49, 7.41 and 7.02, three methyl singlets at $\delta_H$ 2.99, 1.55 and 1.55, and two low-field exchangeable proton singlets at $\delta_H$ 13.78 and 9.63. The $^{13}$C NMR (DEPT) spectrum (Table 2) displayed a total of 21 carbon resonances, including one conjugated ketone carbonyl at $\delta_C$ 182.1, one oxygen-bearing $sp^3$ quaternary carbon at $\delta_C$ 72.7, three methyl signals at $\delta_C$ 50.3 (q, OMe) and 24.8 (2 × q), as well as 16 $sp^2$ carbons. The above NMR spectroscopic features were very similar to those of erysubin A,[4] a prenylated isoflavone also isolated in our current research, the most significant difference being an additional methoxy signal in **1**. The methoxy group should be positioned at C-3″, confirmed

*To whom correspondence should be addressed. E-mail: wangfei@mail.kib.ac.cn (F. Wang); jkliu@mail.kib.ac.cn (J.K. Liu)

**Table 1.** $^{1}$**H NMR spectroscopic data for compounds 1–5 in DMSO-$d_6$ (2.49 ppm)**

| No. | 1 | 2 | 3 | 4 | 5 |
|---|---|---|---|---|---|
| 2 | 8.49 (s) | 8.48 (s) | 8.38 (s) | 7.99 (s) | |
| 6 | | | | | 6.47 (br. s) |
| 8 | 7.41 (s) | 7.36 (s) | 6.57 (s) | 6.38 (s) | 6.57 (br. s) |
| 2′ | 7.41 (d, 8.5) | 7.40 (d, 8.6) | 7.38 (d, 8.6) | 7.26 (d, 8.6) | |
| 3′ | 6.83 (d, 8.5) | 6.83 (d, 8.6) | 6.81 (d, 8.6) | 6.76 (d, 8.6) | 7.11 (s) |
| 5′ | 6.83 (d, 8.5) | 6.83 (d, 8.6) | 6.81 (d, 8.6) | 6.76 (d, 8.6) | |
| 6′ | 7.41 (d, 8.5) | 7.40 (d, 8.6) | 7.38 (d, 8.6) | 7.26 (d, 8.6) | 7.40 (s) |
| 1″ | 7.02 (s) | 7.07 (s) | 5.30 (dd, 7.4, 3.2) | 2.74 (dd, 16.9, 5.5)<br>2.38 (dd, 16.9, 7.4) | |
| 2″ | | | 4.28 (d, 3.2) | 3.62 (ddd, 7.4, 5.5, 4.8) | |
| 4″ | 1.55 (s) | 5.69 (br. s)<br>5.27 (br. s) | 1.16 (s) | 1.27 (s) | |
| 5″ | 1.55 (s) | 2.09 (br. s) | 1.08 (s) | 1.16 (s) | |
| 5-OMe | | | | | 3.82 (s) |
| 5′-OMe | | | | | 3.84 (s) |
| 3″-OMe | 2.99 (s) | | | | |
| 5-OH | 13.78 (s) | 13.79 (s) | 13.41 (s) | | |
| 7-OH | | | | 10.73 (br. s) | * |
| 4′-OH | 9.63 (s) | 9.64 (s) | 9.65 (s) | 9.51 (br. s) | 9.46 (br. s) |
| 1″-OH | | | 5.76 (d, 7.4) | | |
| 2″-OH | | | | 5.19 (d, 4.8) | |
| 3″-OH | | | 4.75 (s) | | |

*Not detected.

by the HMBC correlations from the protons at $\delta_H$ 2.99 (3H, s) and 1.55 (6H, s) to the carbon at $\delta_C$ 72.7 (s, C-3″). The correlations from the protons at $\delta_H$ 7.02 (s, H-1″) and 13.78 (s, 5-OH) to the aromatic quaternary carbon at $\delta_C$ 112.8 (s) verified the connection of the prenyl moiety to C-6. Therefore, the structure of **1** was determined as 3″-*O*-methylerysubin A and named erythrinin D.

Compound **2**, was obtained as a yellow amorphous powder, and its molecular formula was determined to be $C_{20}H_{14}O_5$ based on its ESIMS (pos.): *m/z* 357 $[M + Na]^+$ and HREIMS: *m/z* 334.0842 (calcd for $C_{20}H_{14}O_5$, 334.0841). The $^{1}H$ and $^{13}C$ NMR spectroscopic data (Tables 1 and 2) were very similar to those of erythrinin D (**1**), and contained the characteristic resonances of an isoflavone moiety and a prenyl unit. Major differences from erythrinin D came only from the prenyl unit, and the HMBC correlations from the protons at $\delta_H$ 5.69, 5.27 (each 1H, br. s) and 2.09 (3H, br. s) to the carbon at $\delta_C$ 156.6 (s, C-2″) were indicative of an isopropenyl group at $\alpha$-position of the furan ring. Thus, the structure of **2** was established and named erythrinin E.

Compound **3** was isolated as a white amorphous powder, and with a molecular formula of $C_{20}H_{18}O_7$ on the basis of its ESIMS (pos.): *m/z* 393 $[M + Na]^+$ and HREIMS: *m/z* 370.1042 (calcd for $C_{20}H_{18}O_7$, 370.1053). The $^{1}H$ NMR spectrum (Table 1) displayed six aromatic or olefinic protons at $\delta_H$ 8.38 (1H, s), 7.38 and 6.81 (each 2H, d, $J$ = 8.6 Hz), and 6.57 (1H, s), two vicinal methine protons both attached to oxygen-bearing carbons at $\delta_H$ 5.30 (1H, dd, $J$ = 7.4, 3.2 Hz) and 4.28 (1H, d, $J$ = 3.2 Hz), two methyl singlets at $\delta_H$ 1.16 and 1.08, as well as four exchangeable protons at $\delta_H$ 13.41 (1H, s), 9.65 (1H, s), 5.76 (1H, d, $J$ = 7.4 Hz) and 4.75 (1H, s). The $^{13}C$ NMR (DEPT) spectrum (Table 2) displayed a total of 20 carbon resonances, including 15 characteristic $sp^2$ carbons due to a 5,7,4′-trioxygenated isoflavone nucleus, and a set of carbons at $\delta_C$ 99.6 (d), 69.8 (s), 68.4 (d), 25.8 (q) and 25.1 (q) which most likely originate from a prenyl unit, which could be further characterized as a *trans* 2-(2-hydroxypropan-2-yl)-2,3-dihydrofuran-3-ol moiety compared with the literature data.[5] The connection of the prenyl moiety to C-6 was established by HMBC correlations from the exchangeable protons at $\delta_H$ 5.76 (d, $J$ = 7.4 Hz, 1″-OH) and 13.41 (s, 5-OH) to the aromatic quaternary carbon at $\delta_C$ 112.5 (s, C-6). Since hydroxy proton signals were observed as sharp peaks using DMSO-$d_6$ solvent, their HMBC and ROESY correlations (Figure 1) played an important role in structure elucidation, especially the determination of relative configuration. This isoflavone was isolated in racemic form, in view of a pair of 1:1 ratio chromatographic peaks given by HPLC using a chiral column and specific rotation of zero degrees. Accordingly, the structure of **3** was determined and named (±)-erythrinin F.

**Figure 1.** Key HMBC (arrow) and ROESY (dashed arrow) correlations of erythrinin F (**3**)

Compound **4**, was isolated as a white amorphous powder, with a molecular formula of $C_{20}H_{18}O_6$ according to its ESIMS (pos.): *m/z* 377 $[M + Na]^+$ and HREIMS: *m/z* 354.1099 (calcd for $C_{20}H_{18}O_6$, 354.1103). An analysis of the NMR spectra (Tables 1 and 2) revealed that the compound was also composed of a 5,7,4′-trioxygenated isoflavone nucleus and a prenyl unit. The NMR signals were generally similar to those of eryvarin B,[6] and a prominent difference was that the 3-methylbut-2-en-1-yl signals at C-8 in eryvarin B disappeared in **4** and meanwhile an aromatic methine resonance [$\delta_H$ 6.38 (s), $\delta_C$ 93.6 (d)] re-exposed. The signal missing from the chelated hydroxy proton and a diagnostic up-field shift of the

**Table 2.** $^{13}$**C NMR spectroscopic data for compounds 1–5 in DMSO-*d*$_6$ (39.5 ppm)**

| No. | 1 | 2 | 3 | 4 | 5 |
|---|---|---|---|---|---|
| 2 | 155.1 (d) | 155.2 (d) | 154.3 (d) | 150.2 (d) | 162.6 (s)* |
| 3 | 121.6 (s) | 121.7 (s) | 122.2 (s) | 124.7 (s) | 99.1 (s) |
| 4 | 182.1 (s) | 182.1 (s) | 180.9 (s) | 173.8 (s) | 172.8 (s) |
| 5 | 154.4 (s) | 154.6 (s) | 158.1 (s) | 154.2 (s) | 161.8 (s) |
| 6 | 112.8 (s) | 113.9 (s) | 112.5 (s) | 105.1 (s) | 97.5 (d) |
| 7 | 158.2 (s) | 158.1 (s) | 166.7 (s) | 159.9 (s) | 162.5 (s)* |
| 8 | 90.8 (d) | 90.7 (d) | 88.9 (d) | 93.6 (d) | 96.2 (d) |
| 9 | 153.7 (s) | 154.2 (s) | 158.5 (s) | 157.3 (s) | 156.8 (s) |
| 10 | 106.0 (s) | 106.1 (s) | 105.5 (s) | 107.7 (s) | 106.8 (s) |
| 1′ | 121.0 (s) | 121.1 (s) | 121.1 (s) | 123.1 (s) | 114.1 (s) |
| 2′ | 130.2 (d) | 130.3 (d) | 130.3 (d) | 130.4 (d) | 143.3 (s) |
| 3′ | 115.1 (d) | 115.1 (d) | 115.1 (d) | 114.8 (d) | 99.5 (d) |
| 4′ | 157.4 (s) | 157.5 (s) | 157.5 (s) | 157.0 (s) | 145.5 (s) |
| 5′ | 115.1 (d) | 115.1 (d) | 115.1 (d) | 114.8 (d) | 146.7 (s) |
| 6′ | 130.2 (d) | 130.3 (d) | 130.3 (d) | 130.4 (d) | 103.2 (d) |
| 1″ | 101.3 (d) | 100.6 (d) | 68.4 (d) | 26.1 (t) | |
| 2″ | 160.2 (s) | 156.6 (s) | 99.6 (d) | 66.9 (d) | |
| 3″ | 72.7 (s) | 132.1 (s) | 69.8 (s) | 77.7 (s) | |
| 4″ | 24.8 (q) | 113.9 (t) | 25.8 (q) | 25.5 (q) | |
| 5″ | 24.8 (q) | 18.8 (q) | 25.1 (q) | 20.6 (q) | |
| 5-OMe | | | | | 56.4 (q) |
| 5′-OMe | | | | | 56.4 (q) |
| 3″-OMe | 50.3 (q) | | | | |

*Interchangeable ketone carbon evidenced the ether linkage between C-5 and C-3″. In combination with the detailed HMBC correlations, the isoflavone was characterized as 8-desprenyleryvarin B and given the name erythrinin G. The isoflavone had a high optical purity as documented by HPLC, using a chiral column, and the absolute configuration at C-2″ was postulated as being *R* because it displayed a negative specific rotation value of − 46.0 in MeOH, consistent with those of (*R*)-2,2-dimethylchroman-3-ol derivatives.[7]

Compound **5**, yellow amorphous powder, had a molecular formula of $C_{17}H_{12}O_7$ according to the EIMS: *m/z* 328 $[M]^+$ (100) and HREIMS: *m/z* 328.0587 (calcd for $C_{17}H_{12}O_7$, 328.0583). Considering its biological source, the degree of unsaturation, and the absence of the characteristic proton signal at $\delta_H$ ~ 8.3 (s, H-2) of isoflavone, this compound is most likely a coumaronochromone, which is a special class of isoflavone with an ether linkage between C-2 and C-2′. The $^1$H NMR spectrum (Table 1) displayed four aromatic proton singlets at $\delta_H$ 7.40, 7.11, 6.57 and 6.47, two methoxy signals at $\delta_H$ 3.84 and 3.82, as well as one exchangeable proton at $\delta_H$ 9.46 (br. s), with the $^{13}$C NMR spectrum (Table 2) displaying 15 *sp*$^2$ carbons and two methoxy resonances. The NMR spectroscopic features were very similar to those of desmoxyphyllin A,[8] with the exception of an additional methoxy signal. The signal missing from the chelated hydroxy proton and a diagnostic up-field shift of the ketone carbon indicated methylation of the C-5 hydroxy group. Thereupon, the structure of **5** was established as 7,4′-dihydroxy-5,5′-dimethoxycoumaronochromone and named erythrinin H.

In the mid-1990s, Sato et al.[9] reported several prenylated isoflavones from the fresh pods of *Laburnum anagyroides*. However, the authors proposed an incorrect structure: anagyroidisoflavone A (**6a**). By reconsidering the original NMR spectroscopic data, the structure of **6a** has been revised as 1″-*O*-methylerythrinin F (**6**) (Figure 2), also isolated in our current study and from *Maclura pomifera*.[10] The significant difference with **3** in the $^{13}$C NMR spectrum only arises from the down-field shift (+ 10 ppm) of C-1″ by etherification. The relative configuration of 2-(2-hydroxypropan-2-yl)-2,3-dihydrobenzofuran-3-ol moiety can be established by the diagnostic coupling constants (*trans*-form: ~ 3 Hz; *cis*-form: ~ 6 Hz).[5]

## Experimental Section

**General Experimental Procedures.** Optical rotations were measured on an SGW®-3 (INESA Instrument Co., Ltd., Shanghai, China) automatic polarimeter. UV data were obtained from HPLC online analysis. NMR spectra were carried out on a Bruker Avance III 600 or Bruker DRX-500 (Bruker BioSpin GmbH, Rheinstetten, Germany) spectrometer with deuterated solvent signals used as internal standards. ESI, EI and HREIMS were measured using the Waters Xevo TQ-S and AutoSpec Premier P776 (Waters Corporation, Milford, MA, USA) mass spectrometers, respectively. Silica gel 200–300 mesh (Qingdao Marine Chemical Inc., Qingdao, China) and Sephadex LH-20 (Amersham Biosciences, Uppsala, Sweden) were used for normal pressure column chromatography (CC). Fractions were monitored and analyzed using TLC, in combination with the Agilent 1200 series HPLC system equipped by Extend-C18 column (5 μm, 4.6 × 150 mm). A TCI Chiral MB-S column (5 μm, 4.6 × 250 mm, Tokyo Chemical Industry Co., Ltd., Tokyo, Japan) was applied for determination of enantiomeric purity using the Agilent 1200 series HPLC system.

**Plant Material.** The twigs (~1.5 cm in diameter) of *E. arborescens* were collected from the Pu'er region of Yunnan Province, China, in July 2011, and identified by Mr. Yu Chen of Kunming Institute of Botany, Chinese Academy of Sciences. A voucher specimen (No. BBP0373015EA) was deposited at BioBioPha Co., Ltd.

**Extraction and Isolation.** Dried and powdered twigs (6.0 kg) of *E. arborescens* were extracted with EtOH-$H_2O$ (95:5, v/v; 3 × 12 L, each 5 days) at room temperature. The combined filtrates were concentrated under vacuum to produce a thick, dark extract (*ca.* 340 g), which was fractionated by

**Figure 2.** Structure revision of anagyroidisoflavone A (**6a**)

silica gel CC successively eluted with a gradient of increasing acetone in petroleum ether (PE) (10:1, 7:1, 5:1, 3:1, 2:1, 1:1, 0:1; v/v) and MeOH to produce fractions A–H, respectively. The fraction D was repeatedly separated on silica gel ($CHCl_3$/MeOH; 100:0→20:1) and Sephadex LH-20 CC ($CHCl_3$/MeOH; 1:1) to yield **2** (9 mg) and **1** (48 mg). The fraction E was further isolated on silica gel ($CHCl_3$/MeOH; 80:1→20:1) and by slow evaporation from $CHCl_3$/MeOH to yield **5** (3 mg). Compounds **3** (22 mg) and **4** (63 mg) were eventually acquired by means of repeated silica gel ($CHCl_3$/MeOH; 50:1→10:1) and Sephadex LH-20 CC ($CHCl_3$/MeOH; 1:1) from the fraction F. The retention times ($t_R$) of **1**–**5** on an analytical HPLC Extend-C18 column (20%→100% MeOH in $H_2O$ over 8.0 min followed by 100% MeOH to 13 min, 1.0 ml/min, 20 °C) were 9.5, 10.6, 7.1, 6.7, and 7.8 min, respectively.

**Erythrinin D (1):** yellow amorphous powder; UV (MeOH) $\lambda_{max}$: 266, 350 nm; $^1$H NMR data: see Table 1; $^{13}$C NMR data: see Table 2; ESIMS (pos.): *m/z* 389 $[M + Na]^+$; HREIMS: *m/z* 366.1099 (calcd for $C_{21}H_{18}O_6$, 366.1103).

**Erythrinin E (2):** yellow amorphous powder; UV (MeOH) $\lambda_{max}$: 284, 322 (sh), 354 nm; $^1$H NMR data: see Table 1; $^{13}$C NMR data: see Table 2; ESIMS (pos.): *m/z* 357 $[M + Na]^+$; HREIMS: *m/z* 334.0842 (calcd for $C_{20}H_{14}O_5$, 334.0841).

**(±)-Erythrinin F (3):** white amorphous powder, $[\alpha]_D^{28}$ 0 (*c* 0.05, MeOH); Chiral HPLC analysis of the enantiomers: $t_{R(a)}$ = 4.89 min, $t_{R(b)}$ = 5.15 min (20% MeCN in $H_2O$ over 10.0 min, 1.0 ml/min, 25 °C); UV (MeOH) $\lambda_{max}$: 262 nm; $^1$H NMR data: see Table 1; $^{13}$C NMR data: see Table 2; ESIMS (pos.): *m/z* 393 $[M + Na]^+$; HREIMS: *m/z* 370.1042 (calcd for $C_{20}H_{18}O_7$, 370.1053).

**Erythrinin G (4):** white amorphous powder, $[\alpha]_D^{30}$ − 46.0 (*c* 0.05, MeOH); UV (MeOH) $\lambda_{max}$: 260, 324 (sh) nm; $^1$H NMR data: see Table 1; $^{13}$C NMR data: see Table 2; ESIMS (pos.): *m/z* 377 $[M + Na]^+$; HREIMS: *m/z* 354.1099 (calcd for $C_{20}H_{18}O_6$, 354.1103).

**Erythrinin H (5):** yellow amorphous powder; UV (MeOH) $\lambda_{max}$: 254, 280, 306, 326 nm; $^1$H NMR data: see Table 1; $^{13}$C NMR data: see Table 2; EIMS: *m/z* 328 $[M]^+$ (100), 311 (31), 299 (21), 283 (43), 282 (40), 270 (14), 267 (18); HREIMS: *m/z* 328.0587 (calcd for $C_{17}H_{12}O_7$, 328.0583).

**(±)-1″-*O*-Methylerythrinin F ( = anagyroidisoflavone A, 6):** white amorphous powder, $[\alpha]_D^{28}$ 0 (*c* 0.05, MeOH); UV (MeOH) $\lambda_{max}$: 212, 263 nm; $^1$H NMR ($CD_3OD$): $\delta_H$ 8.10 (1H, s, H-2), 6.46 (1H, s, H-8), 7.37 (2H, d, *J* = 8.6 Hz, H-2′ and H-6′), 6.83 (2H, d, *J* = 8.6 Hz, H-3′ and H-5′), 5.15 (1H, d, *J* = 2.5 Hz, H-1″), 4.46 (1H, d, *J* = 2.5 Hz, H-2″), 1.27 (3H, s, H-4″), 1.16 (3H, s, H-5″), 3.53 (3H, s, OMe); $^{13}$C NMR ($CD_3OD$): $\delta_C$ 155.0 (d, C-2), 124.8 (s, C-3), 182.8 (s, C-4), 160.5 (s, C-5), 110.9 (s, C-6), 168.9 (s, C-7), 90.2 (d, C-8), 161.0 (s, C-9), 107.2 (s, C-10), 123.0 (s, C-1′), 131.4 (2 × d, C-2′ and C-6′), 116.3 (2 × d, C-3′ and C-5′), 158.9 (s, C-4′), 79.8 (d, C-1″), 98.1 (d, C-2″), 71.7 (s, C-3″), 26.1 (q, C-4″), 24.6 (q, C-5″), 57.3 (q, OMe).

## Electronic Supplementary Material

Supplementary material is available in the online version of this article at http://dx.doi.org/10.1007/s13659-013-0062-3 and is accessible for authorized users.

## Acknowledgments

This work was financially supported by the "Large-scale Compound Library" project of the National Development and Reform Commission of China, the National Basic Research Program of China (973 Program) 2009CB522300, and the "Western Light" talents training program of Chinese Academy of Sciences.

**Open Access** This article is distributed under the terms of the Creative Commons Attribution License which permits any use, distribution, and reproduction in any medium, provided the original author(s) and source are credited.

## Electronic supplementary material

Supplementary material, approximately 1.62 MB.
