# Supplementary material for: New isoflavonoids from Erythrina arborescens and structure revision of anagyroidisoflavone A
Source: Nat Prod Bioprospect. 2013 Oct 14;3(5):238–42. doi: 10.1007/s13659-013-0062-3 (PMC4131621; doi:10.1007/s13659-013-0062-3)

Electronic Supplementary Material

New isoflavonoids from *Erythrina arborescens* and structure revision of anagyroidisoflavone A

Fei WANG,[a,b,*] Xu-Long LI,[a] Guo-Zhu WEI,[a] Fu-Cai REN,[a] and Ji-Kai LIU[b,*]

[a]BioBioPha Co., Ltd., Kunming 650201, China

[b]State Key Laboratory of Phytochemistry and Plant Resources in West China, Kunming Institute of Botany, Chinese Academy of Sciences, Kunming 650201, China

Received 9 August 2013; Accepted 15 September 2013

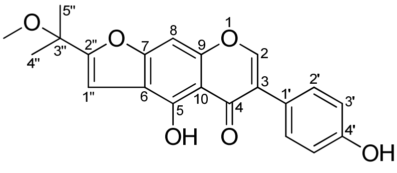

1

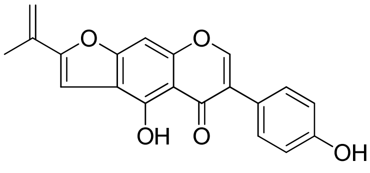

2

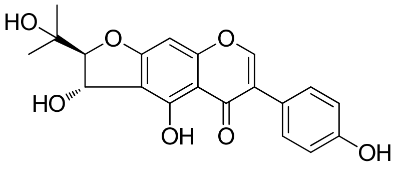

3

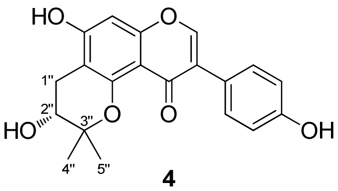

4

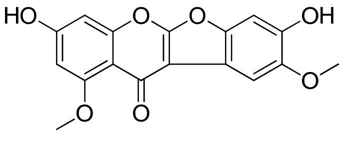

5

Structures of compounds **1**–**5**

*To whom correspondence should be addressed. E-mail: wangfei@mail.kib.ac.cn (F. Wang); jkliu@mail.kib.ac.cn (J.K. Liu)

Content list:

S1. ^{1}H NMR spectrum (500 MHz, DMSO-d_6) of erythrinin D (**1**).

S2. ^{13}C NMR spectrum (125 MHz, DMSO-d_6) of erythrinin D (**1**).

S3. HMBC spectrum (500 MHz, DMSO-d_6) of erythrinin D (**1**).

S4. ^{1}H NMR spectrum (500 MHz, DMSO-d_6) of erythrinin E (**2**).

S5. ^{13}C NMR spectrum (100 MHz, DMSO-d_6) of erythrinin E (**2**).

S6. HMBC spectrum (500 MHz, DMSO-d_6) of erythrinin E (**2**).

S7. ^{1}H NMR spectrum (600 MHz, DMSO-d_6) of erythrinin F (**3**).

S8. ^{13}C NMR spectrum (150 MHz, DMSO-d_6) of erythrinin F (**3**).

S9. HMBC spectrum (600 MHz, DMSO-d_6) of erythrinin F (**3**).

S10. ROESY spectrum (600 MHz, DMSO-d_6) of erythrinin F (**3**).

S11. ^{1}H NMR spectrum (600 MHz, DMSO-d_6) of erythrinin G (**4**).

S12. ^{13}C NMR spectrum (150 MHz, DMSO-d_6) of erythrinin G (**4**).

S13. HMBC spectrum (600 MHz, DMSO-d_6) of erythrinin G (**4**).

S14. ^{1}H NMR spectrum (500 MHz, DMSO-d_6) of erythrinin H (**5**).

S15. ^{13}C NMR spectrum (100 MHz, DMSO-d_6) of erythrinin H (**5**).

S16. HMBC spectrum (500 MHz, DMSO-d_6 + TFA) of erythrinin H (**5**).

S17. HSQC spectrum (500 MHz, DMSO-d_6 + TFA) of erythrinin H (**5**).

S18. Chiral HPLC analysis of the enantiomeric purity of erythrinin F (**3**).

S19. HPLC analysis and its UV spectrum of erythrinin D (**1**).
S20. HPLC analysis and its UV spectrum of erythrinin E (**2**).
S21. HPLC analysis and its UV spectrum of erythrinin F (**3**).
S22. HPLC analysis and its UV spectrum of erythrinin G (**4**).
S23. HPLC analysis and its UV spectrum of erythrinin H (**5**).
S24. HPLC analysis and its UV spectrum of 1″-*O*-methylerythrinin F (**6**).

S1. ^{1}H NMR spectrum (500 MHz, DMSO-d_6) of erythrinin D (**1**).

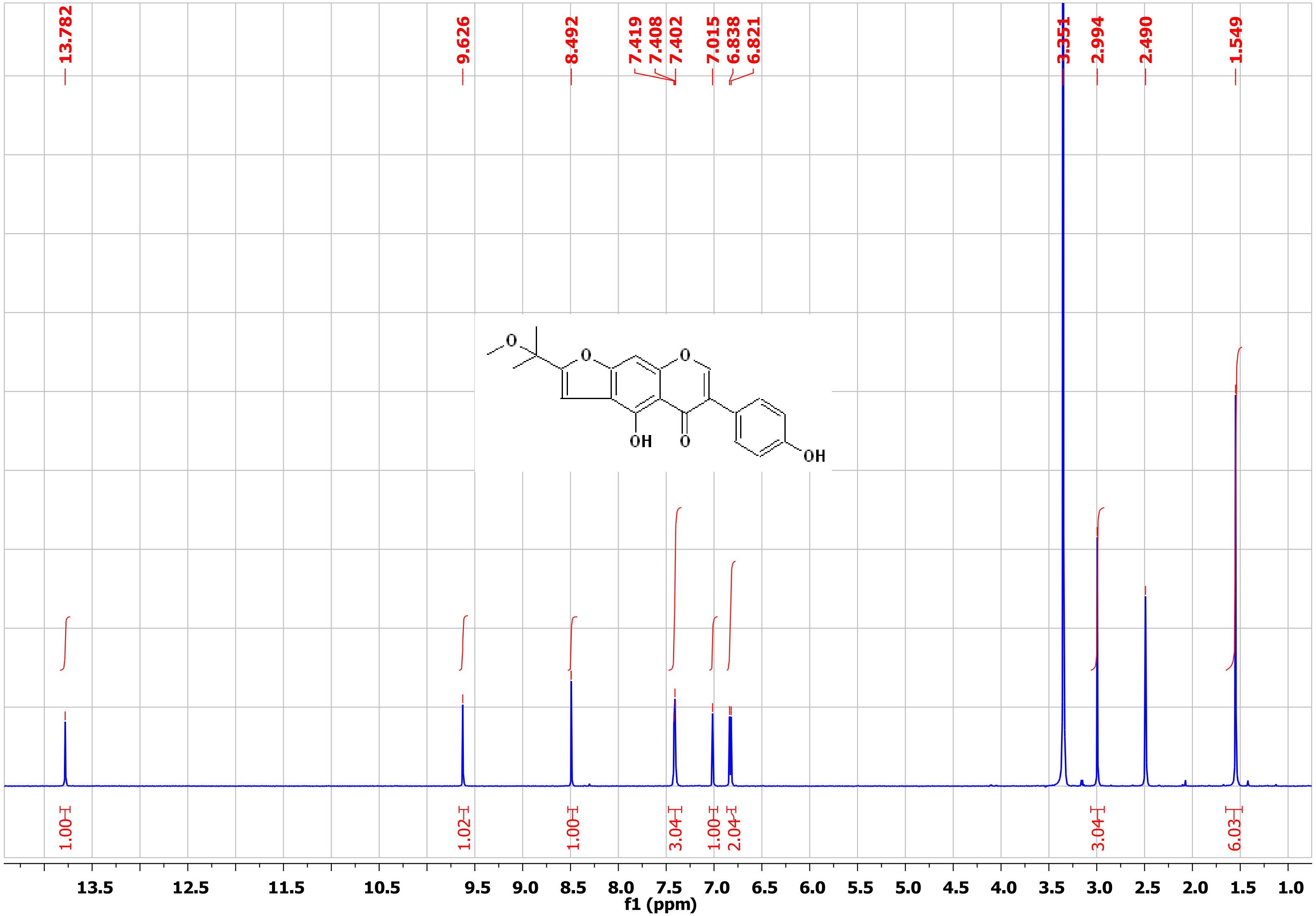

S2. ^{13}C NMR spectrum (125 MHz, DMSO-d_6) of erythrinin D (**1**).

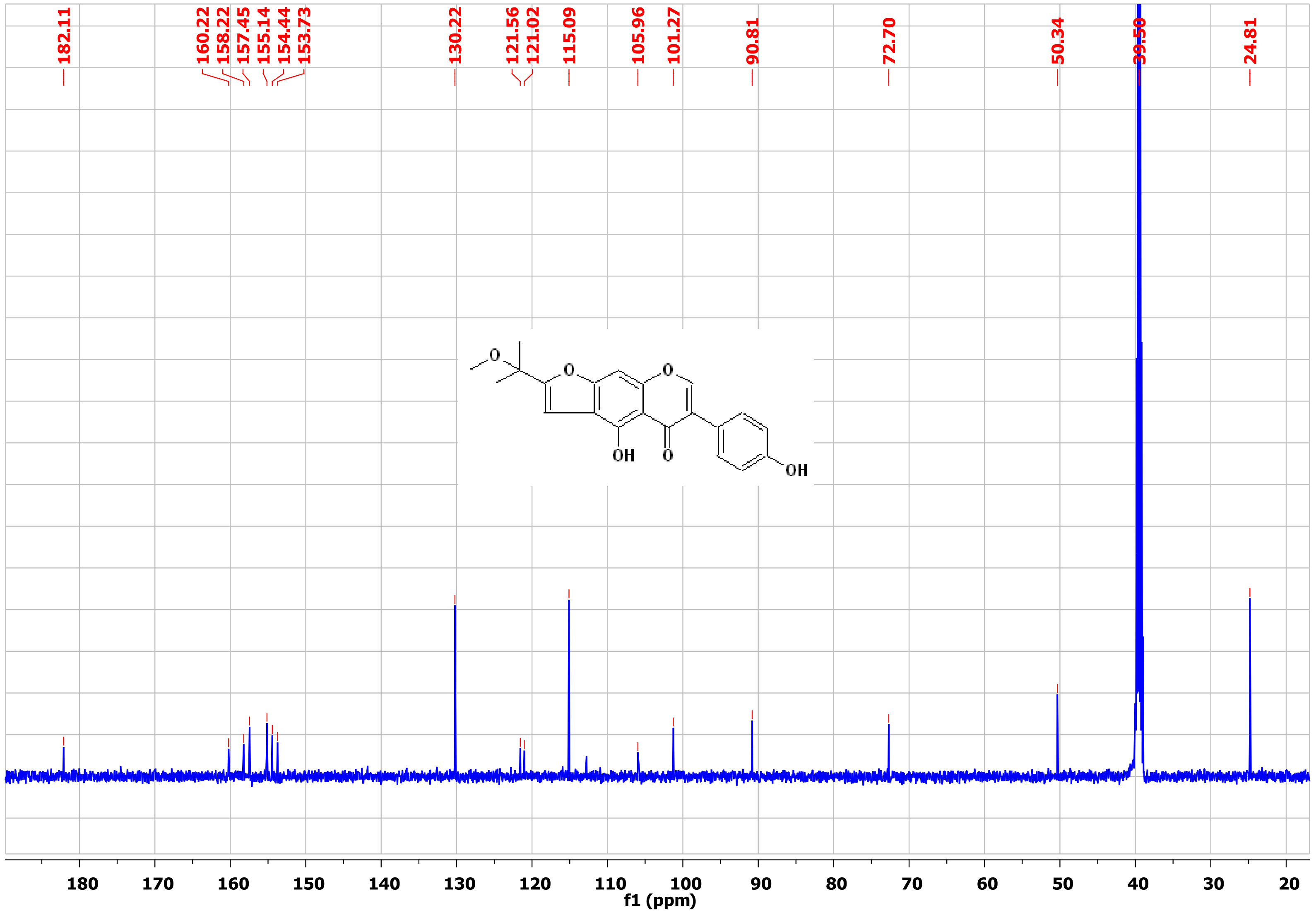

S3. HMBC spectrum (500 MHz, DMSO-d_6) of erythrinin D (**1**).

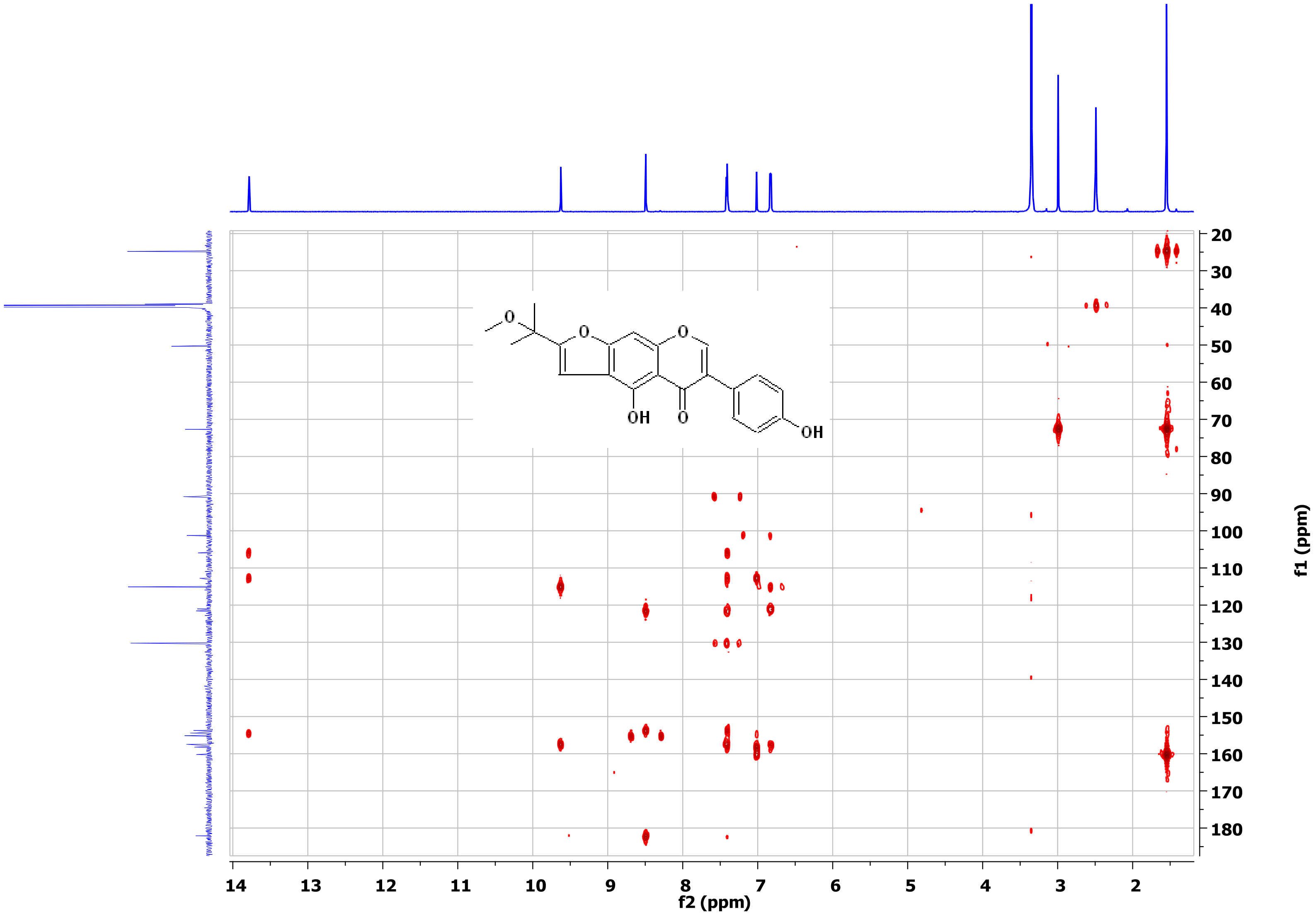

S4. ^{1}H NMR spectrum (500 MHz, DMSO-d_6) of erythrinin E (**2**).

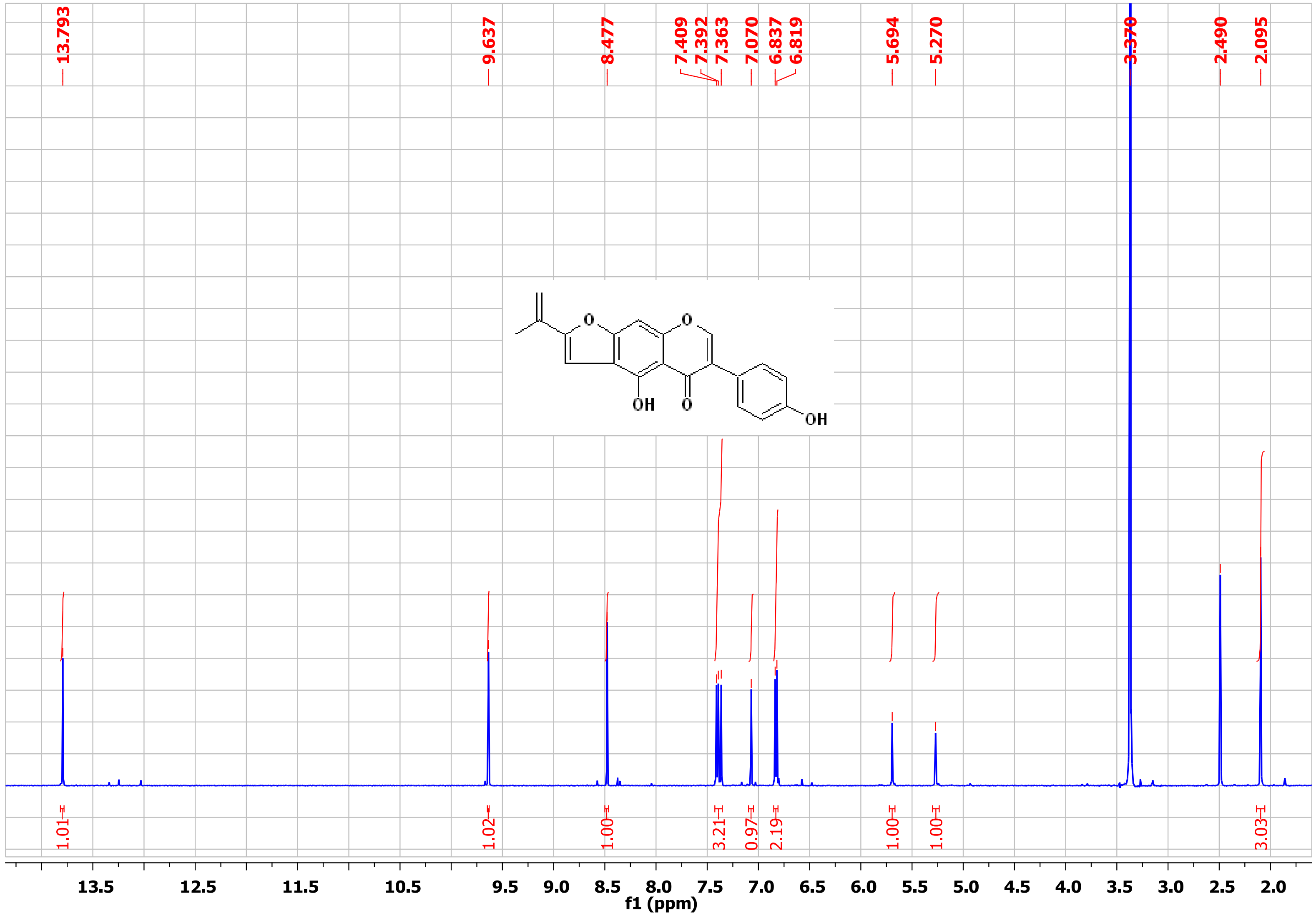

S5. ^{13}C NMR spectrum (100 MHz, DMSO-d_6) of erythrinin E (**2**).

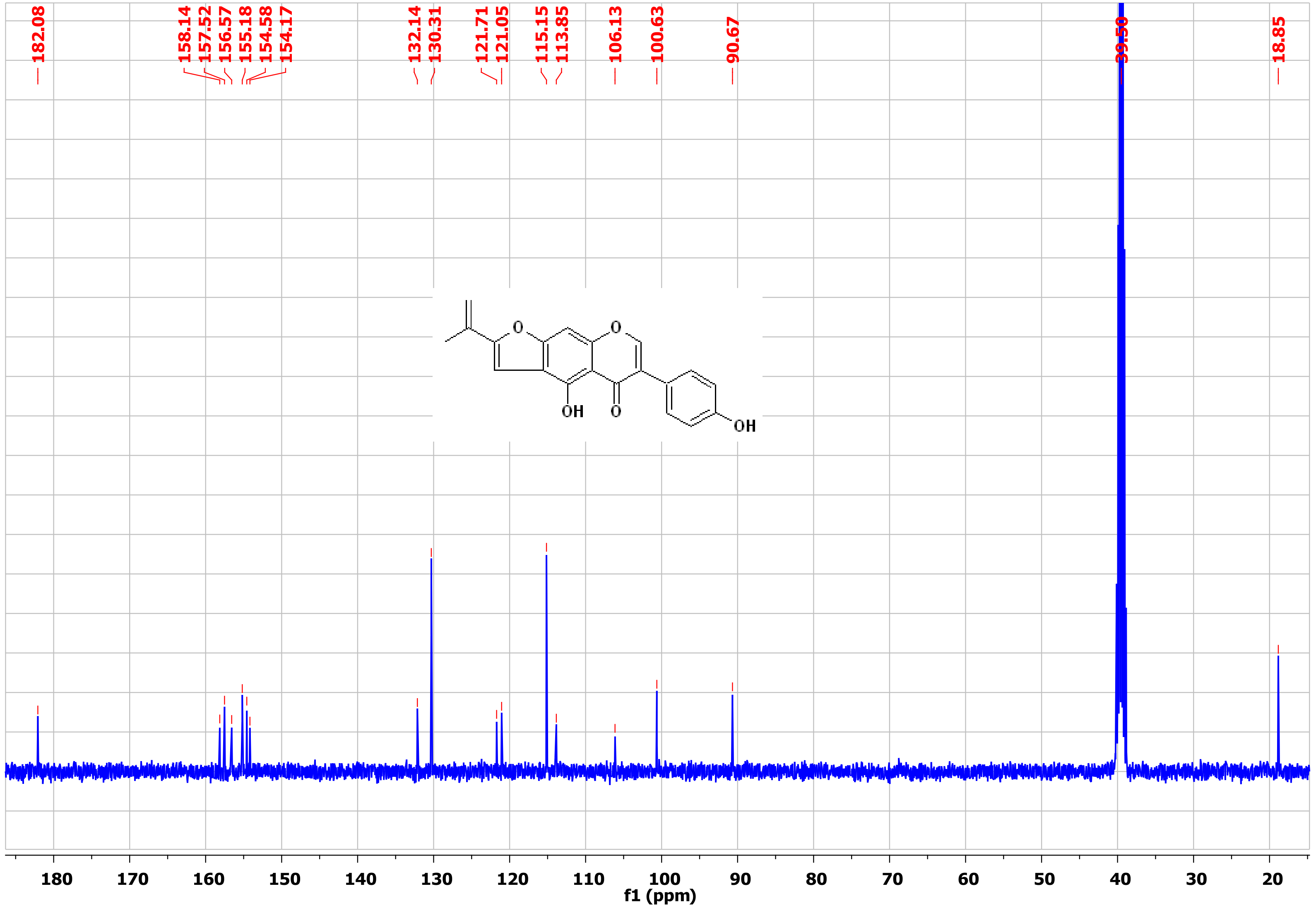

S6. HMBC spectrum (500 MHz, DMSO-d_6) of erythrinin E (**2**).

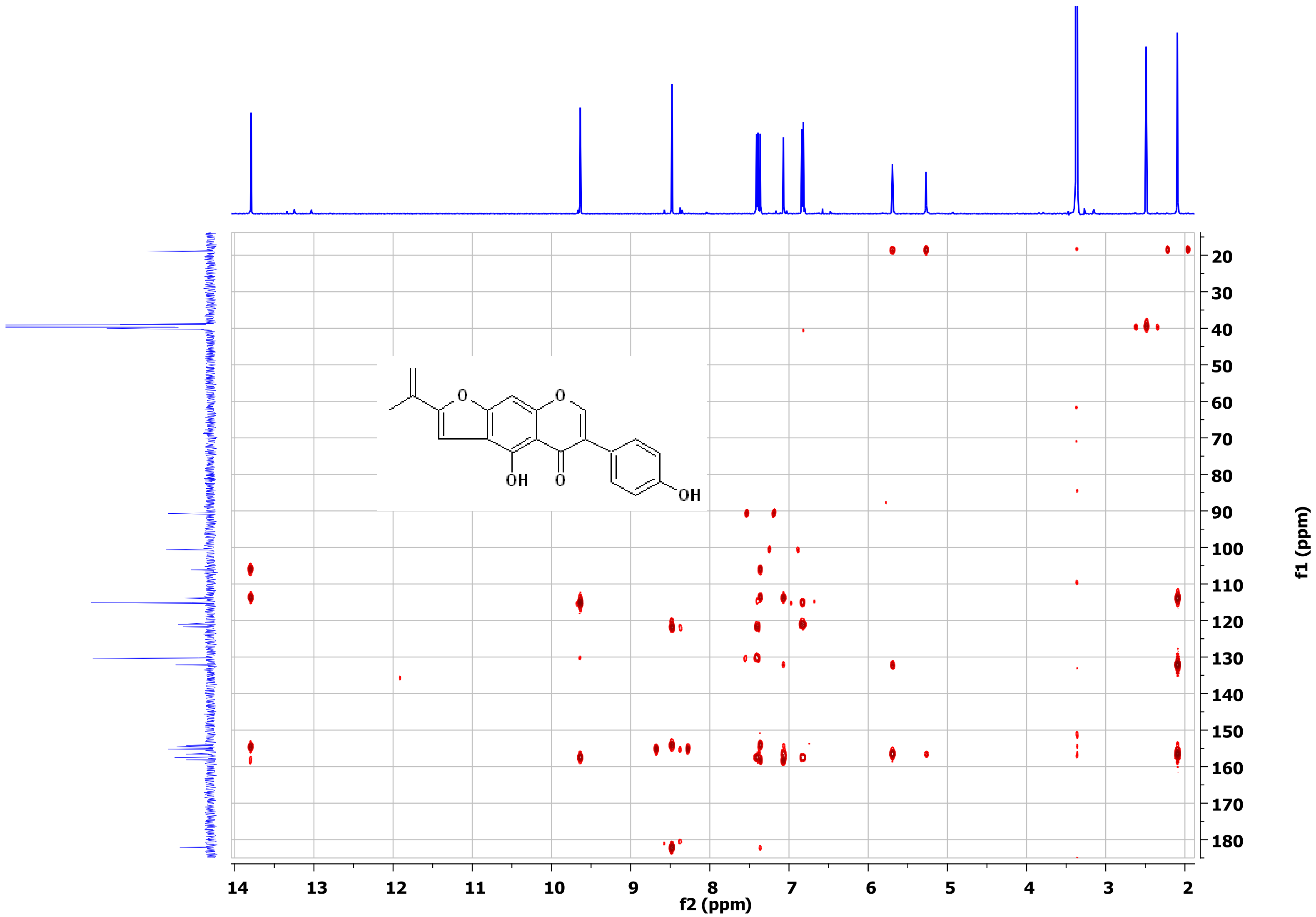

S7. ^{1}H NMR spectrum (600 MHz, DMSO-d_6) of erythrinin F (**3**).

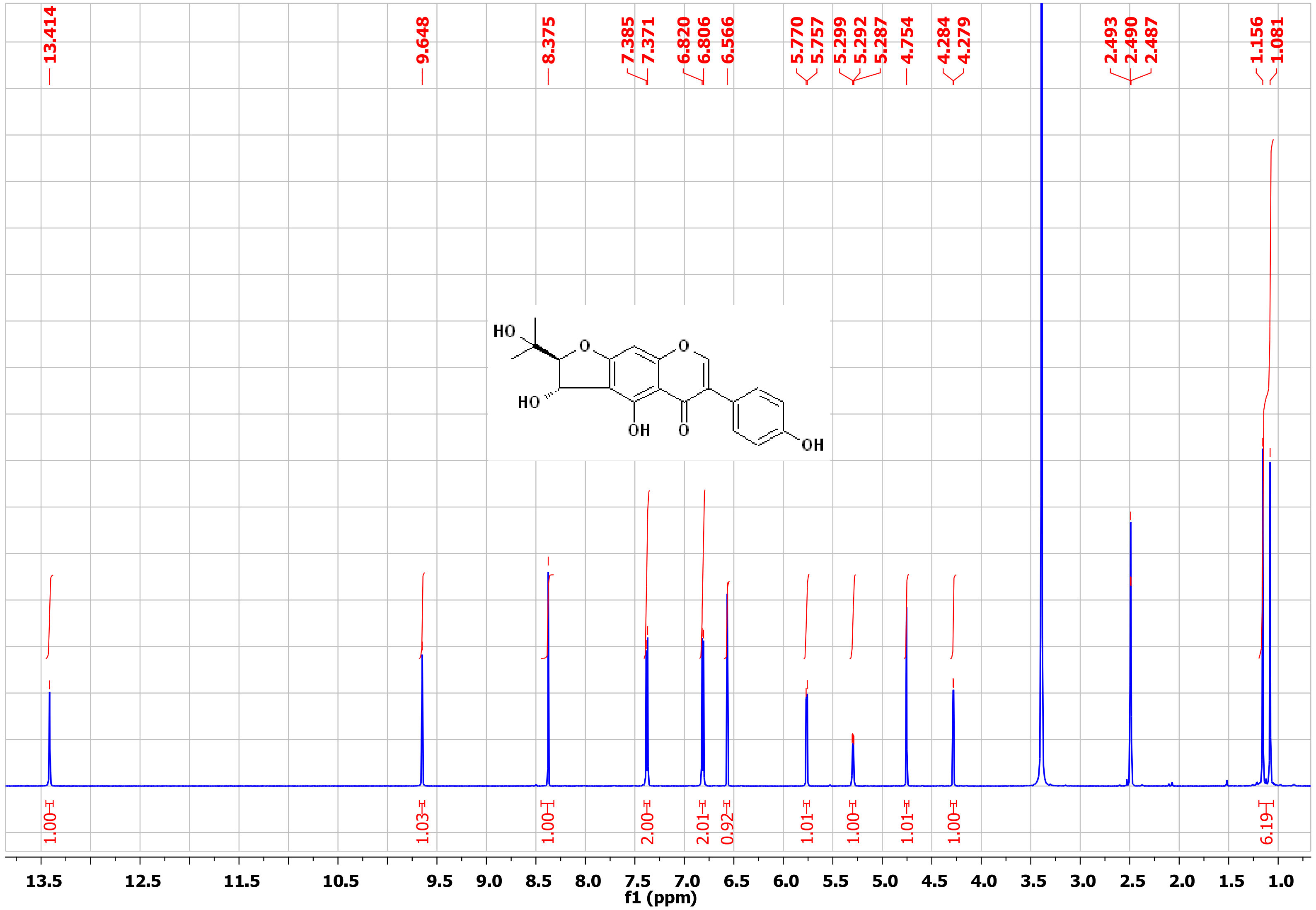

S8. ^{13}C NMR spectrum (150 MHz, DMSO-d_6) of erythrinin F (**3**).

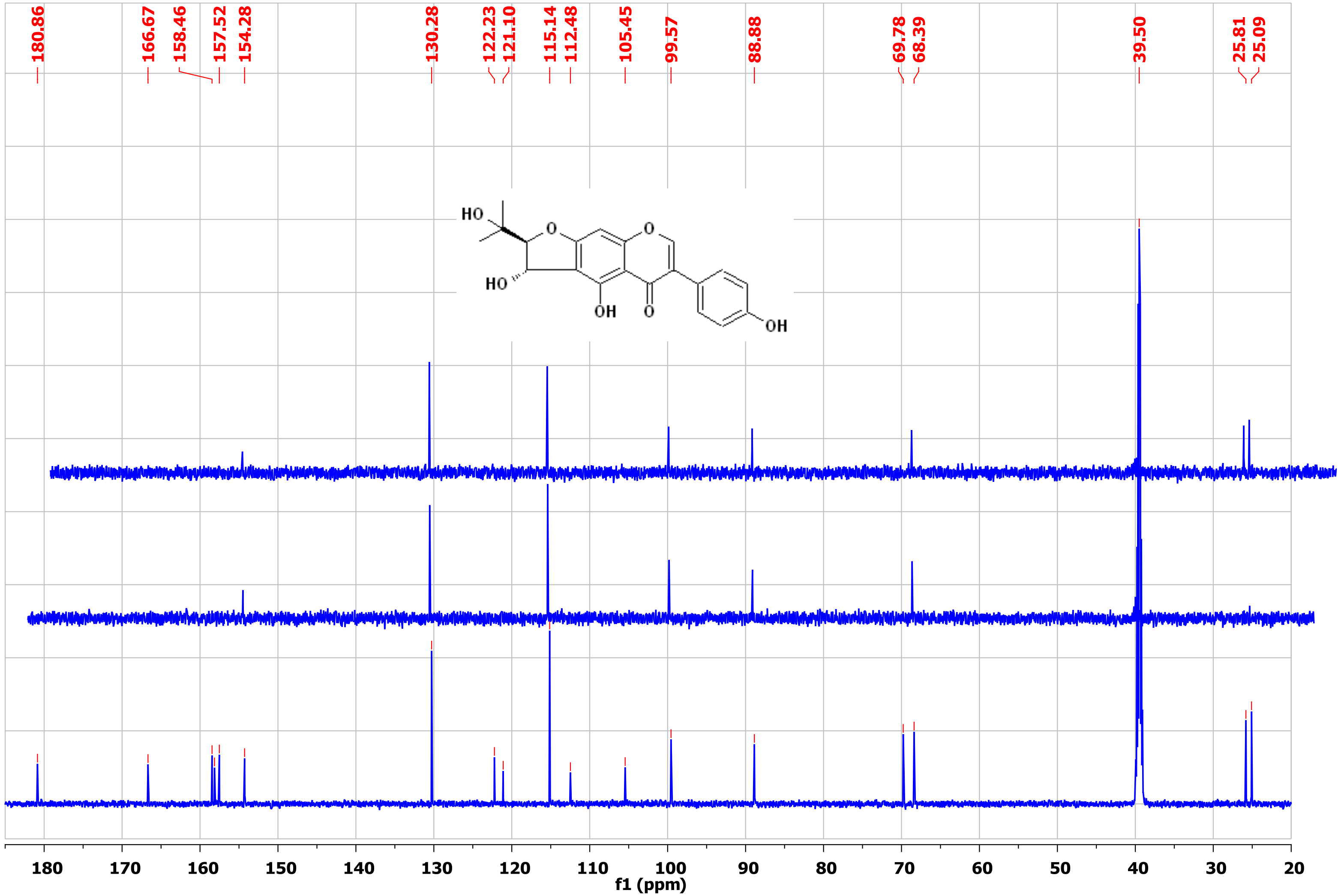

S9. HMBC spectrum (600 MHz, DMSO-d_6) of erythrinin F (**3**).

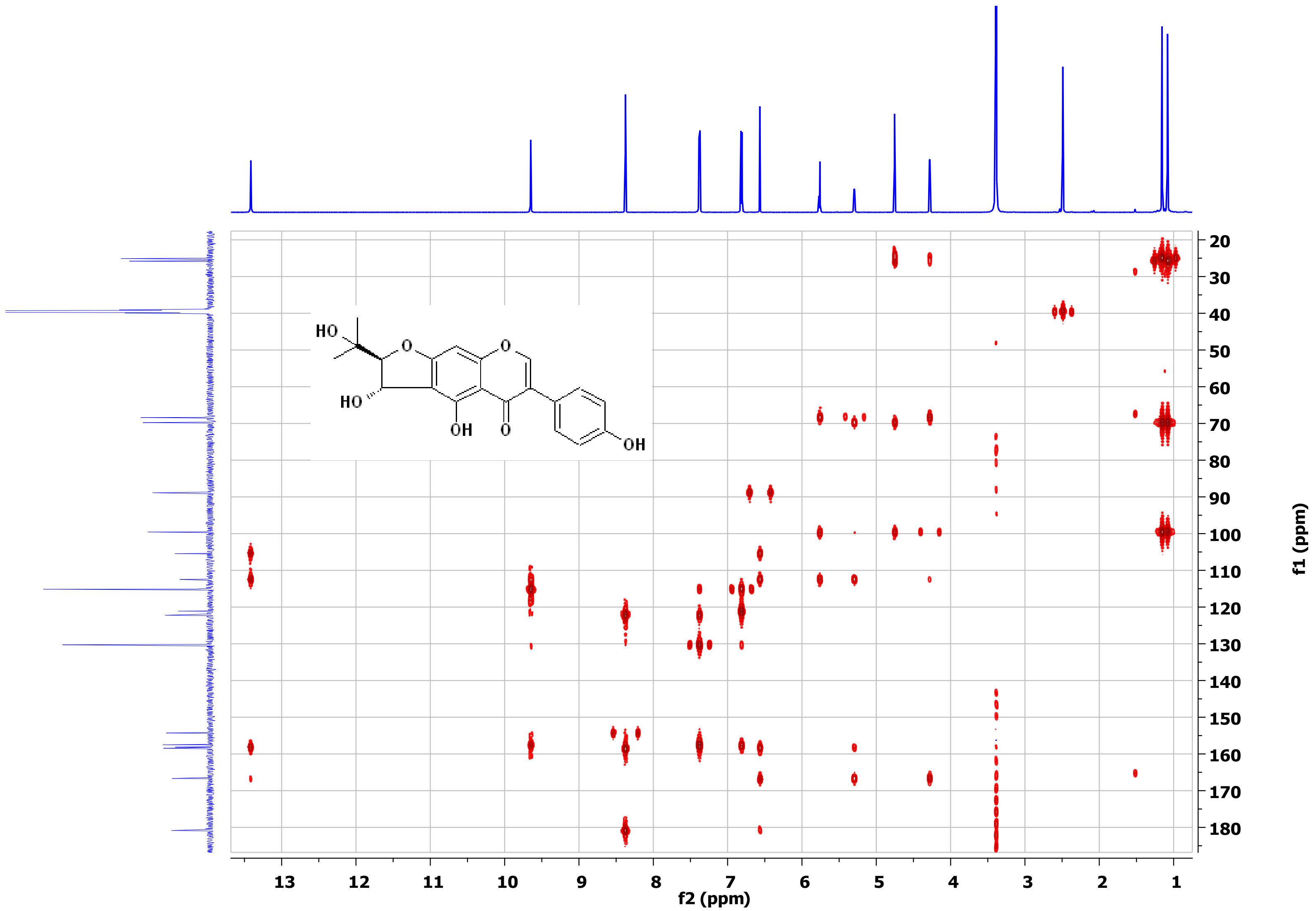

S10. ROESY spectrum (600 MHz, DMSO-d_6) of erythrinin F (**3**).

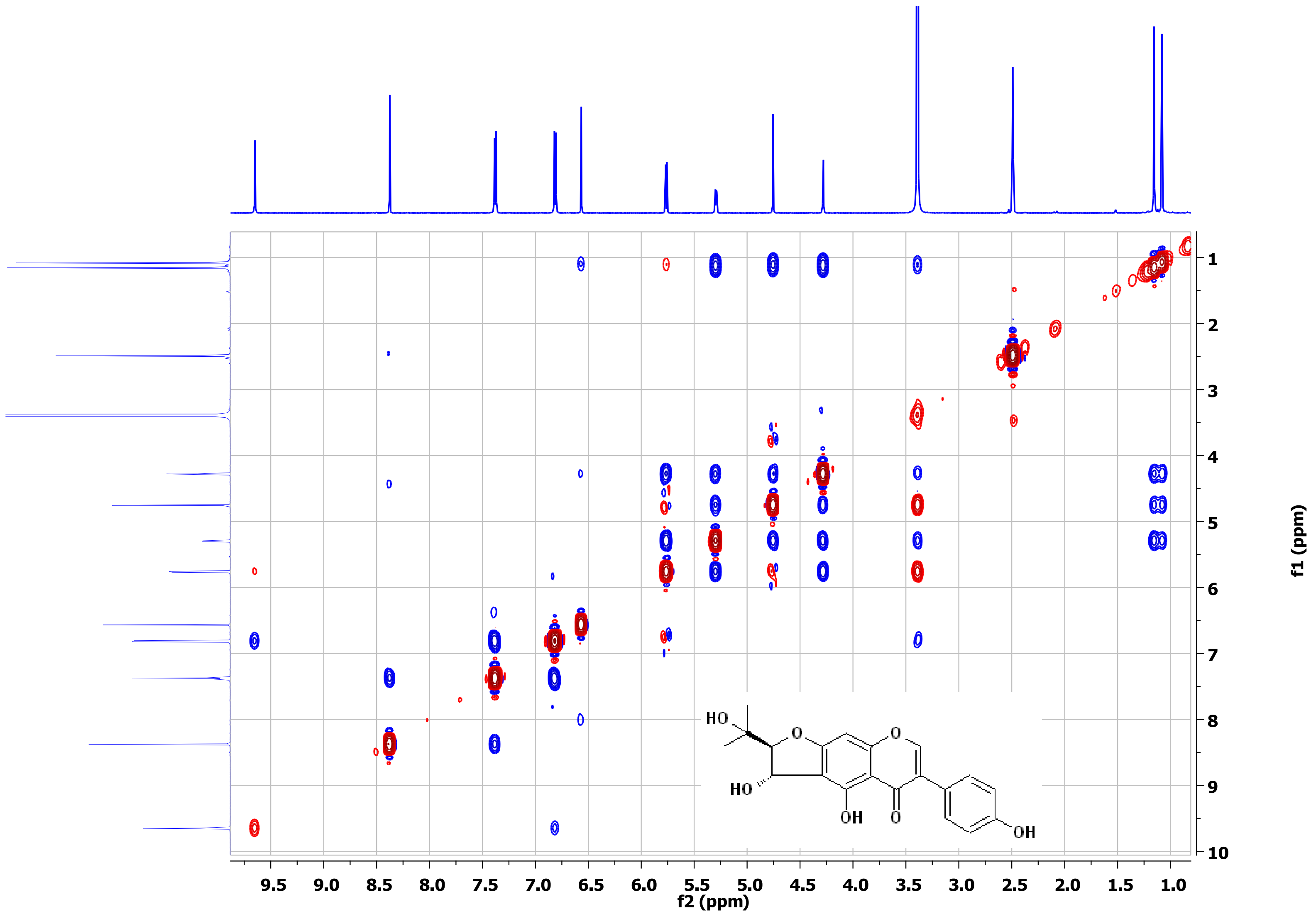

S11. ^{1}H NMR spectrum (600 MHz, DMSO-d_6) of erythrinin G (**4**).

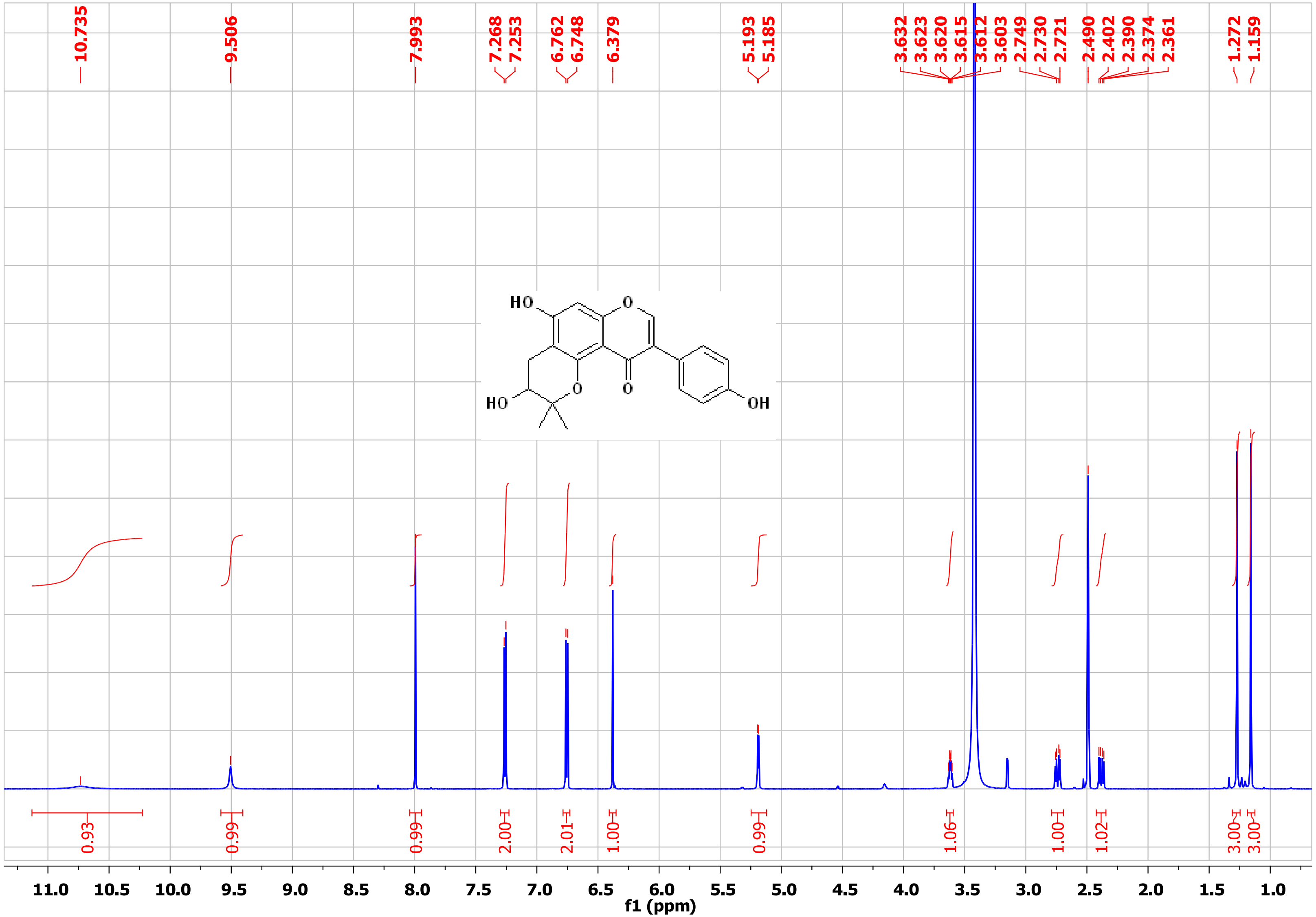

S12. ^{13}C NMR spectrum (150 MHz, DMSO-d_6) of erythrinin G (**4**).

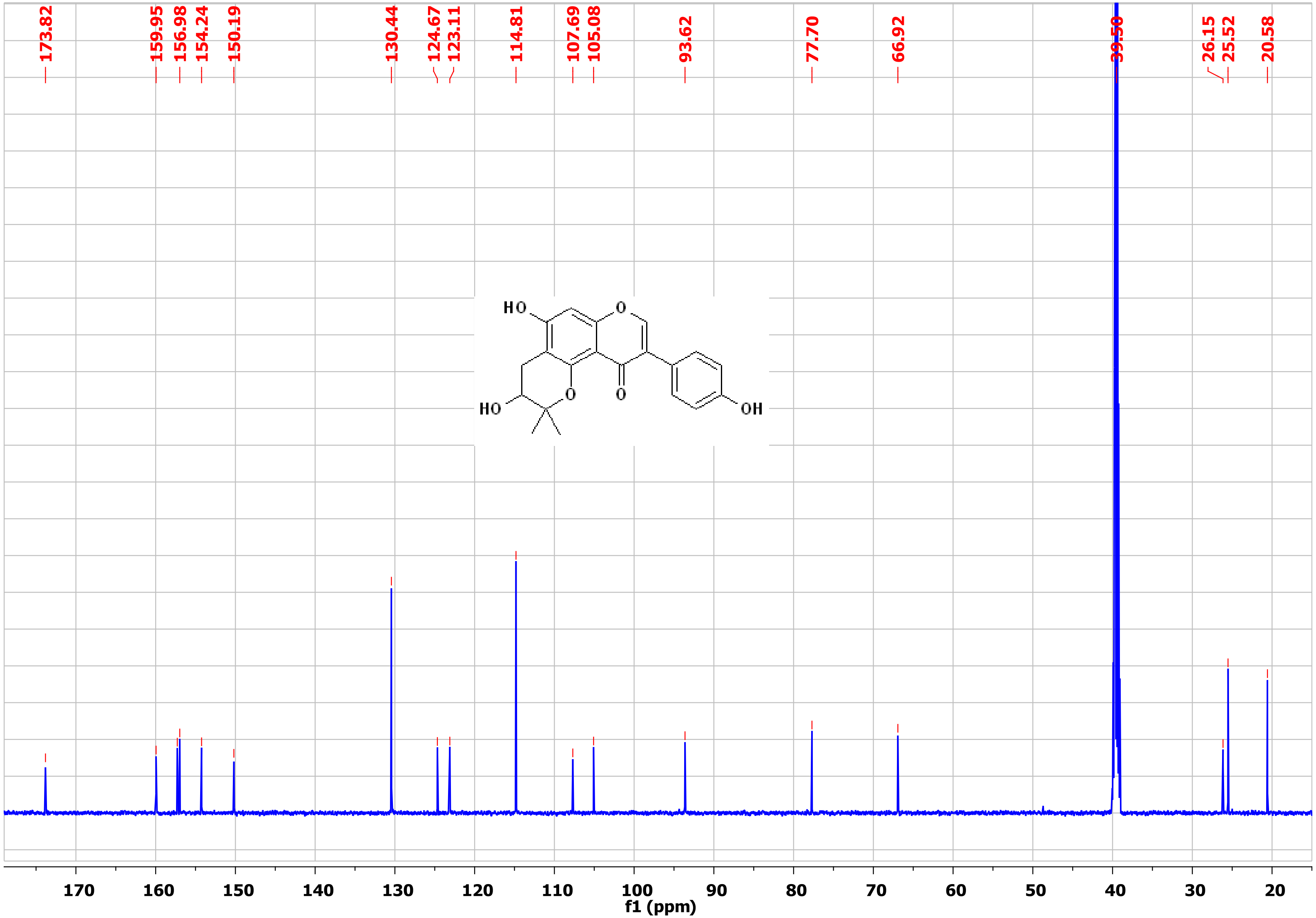

S13. HMBC spectrum (600 MHz, DMSO-d_6) of erythrinin G (**4**).

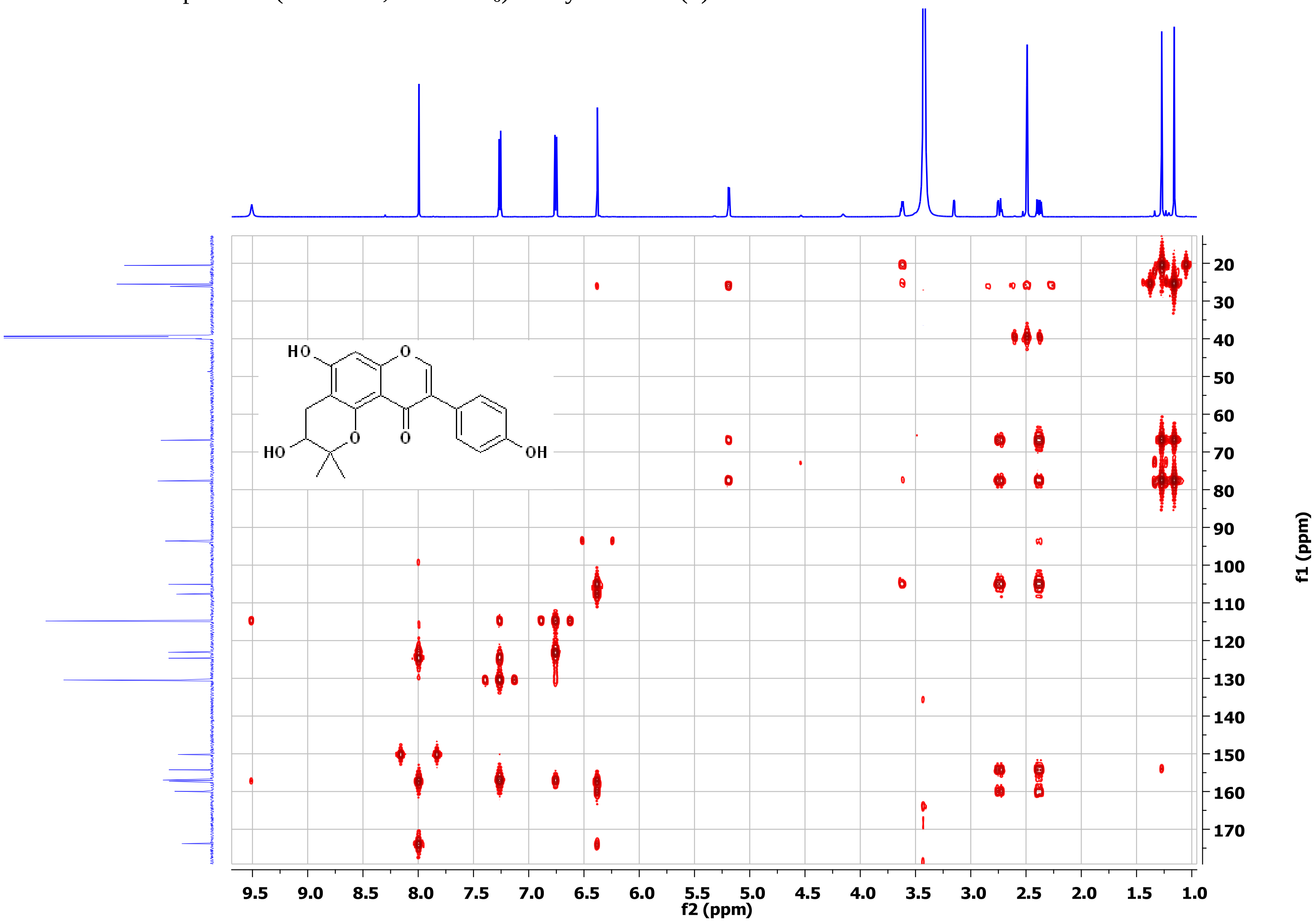

S14. ^{1}H NMR spectrum (500 MHz, DMSO-d_6) of erythrinin H (**5**).

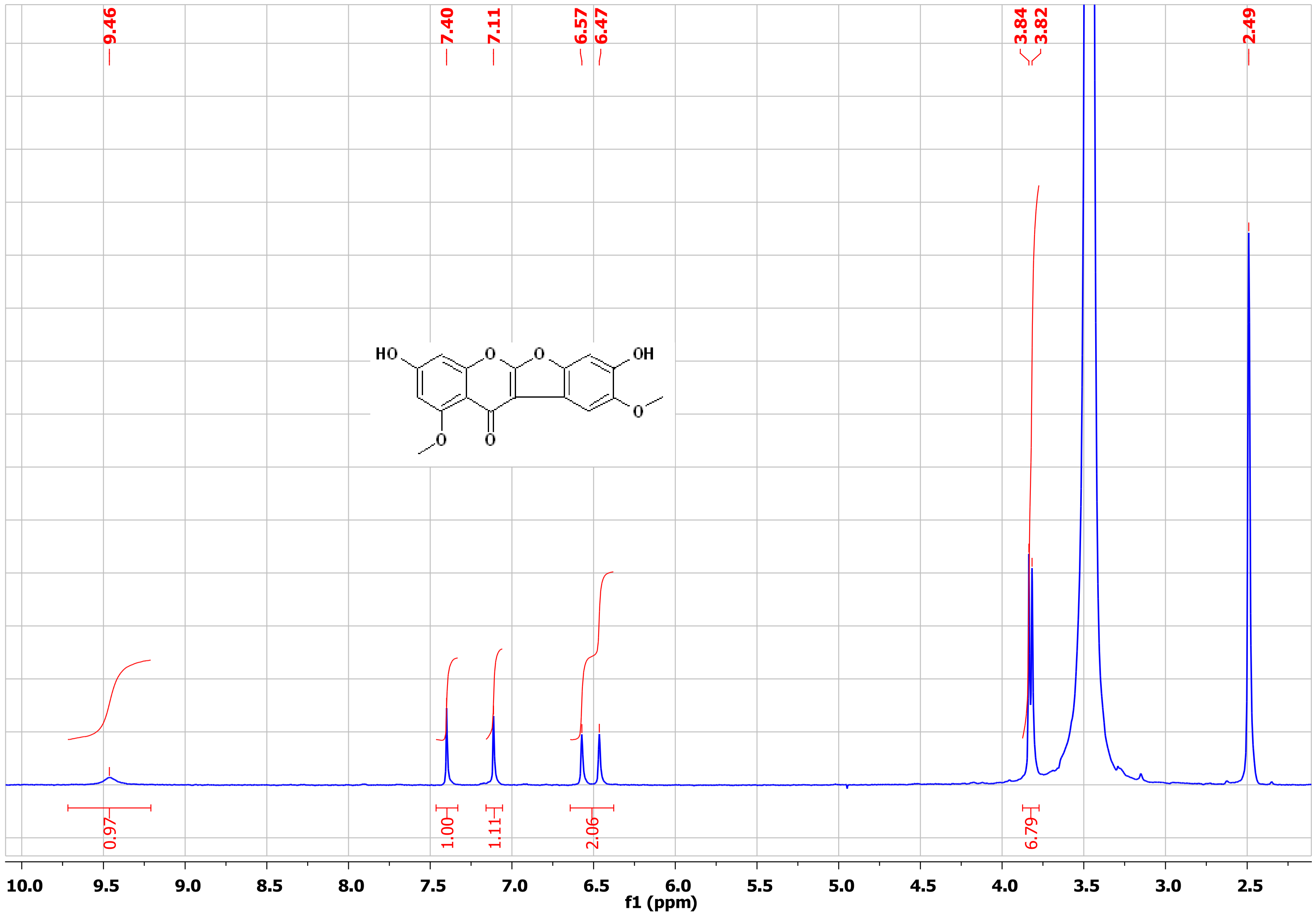

S15. ^{13}C NMR spectrum (100 MHz, DMSO-d_6) of erythrinin H (**5**).

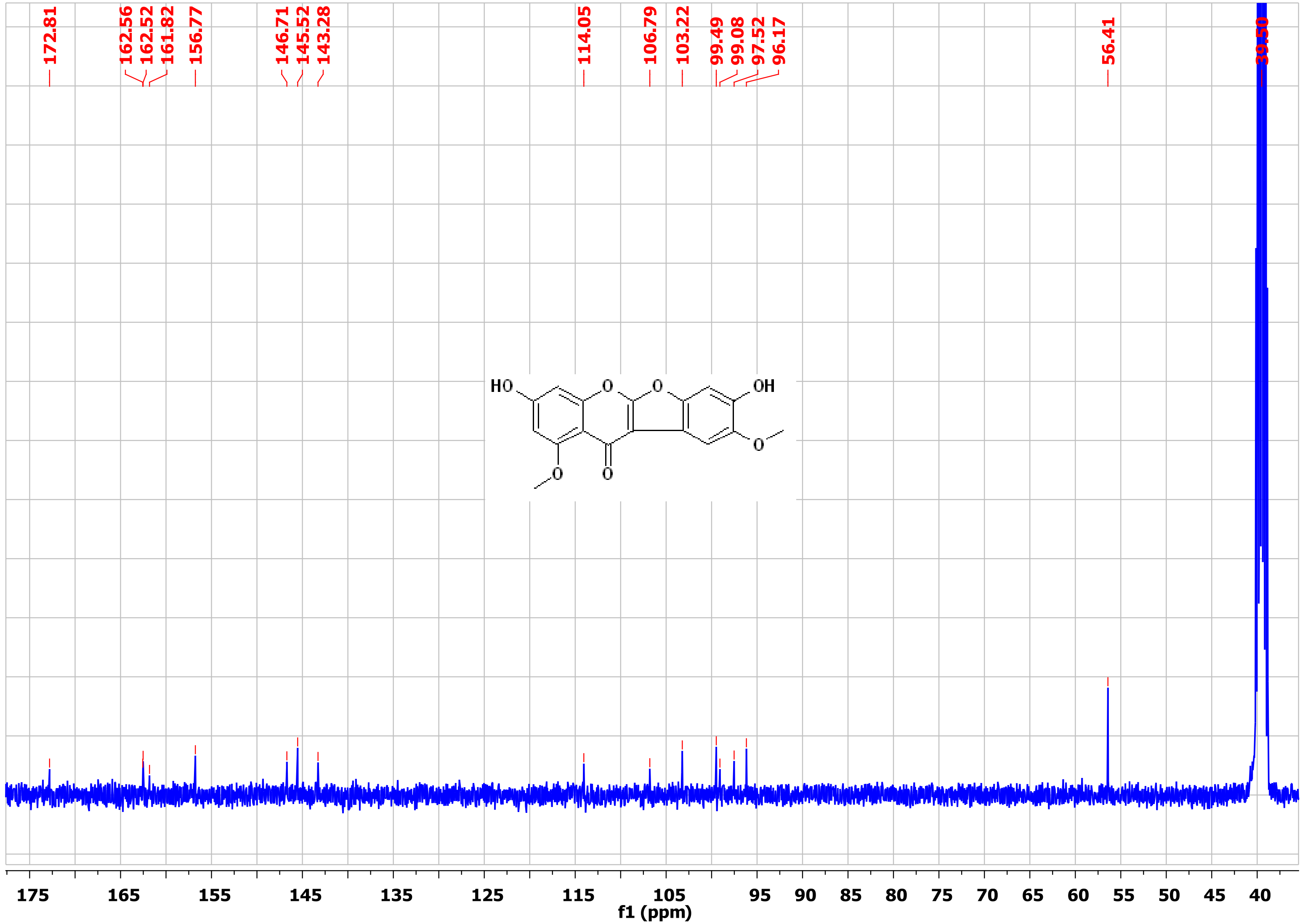

S16. HMBC spectrum (500 MHz, DMSO-d_6 + TFA) of erythrinin H (**5**).

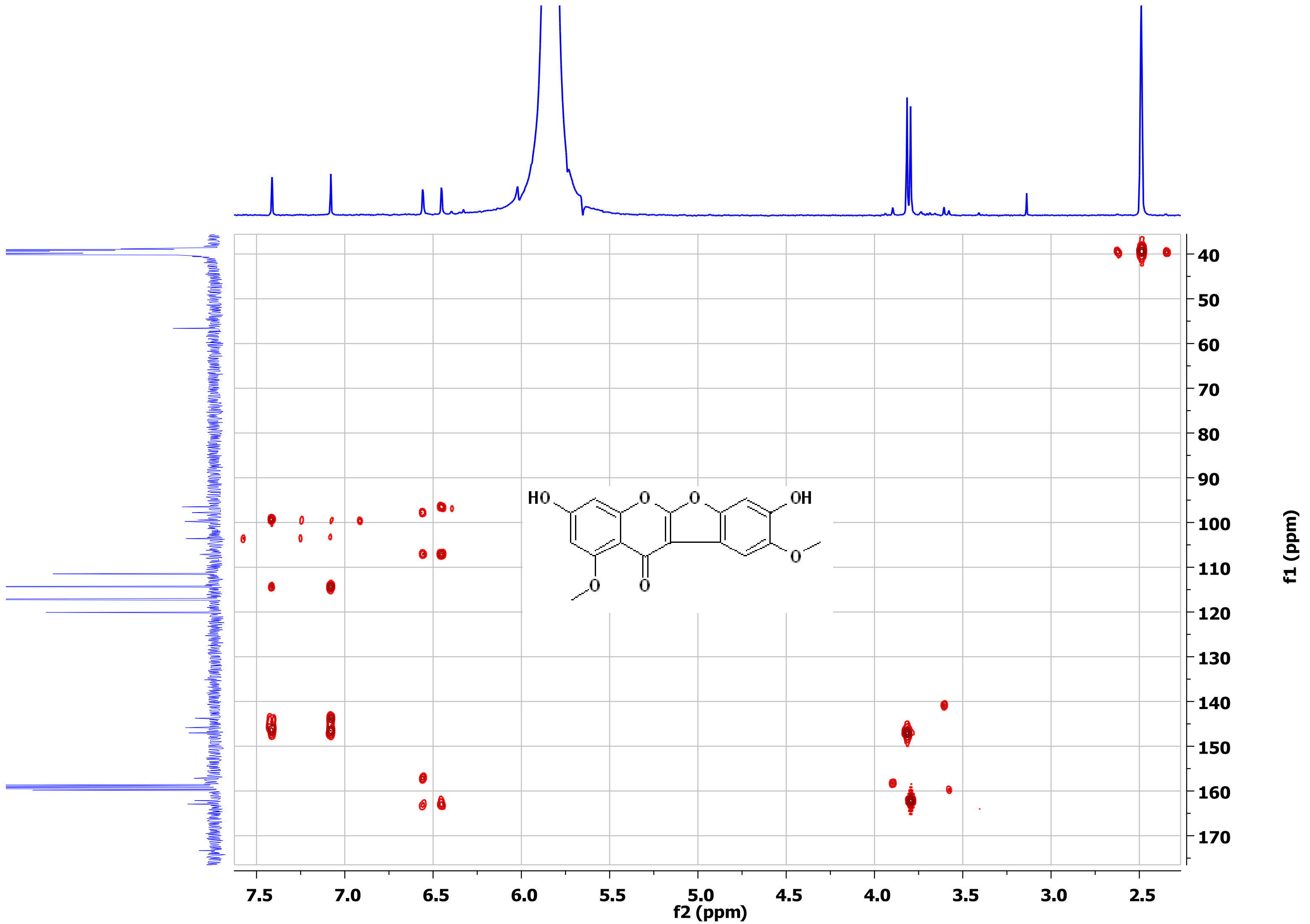

S17. HSQC spectrum (500 MHz, DMSO-d_6 + TFA) of erythrinin H (**5**).

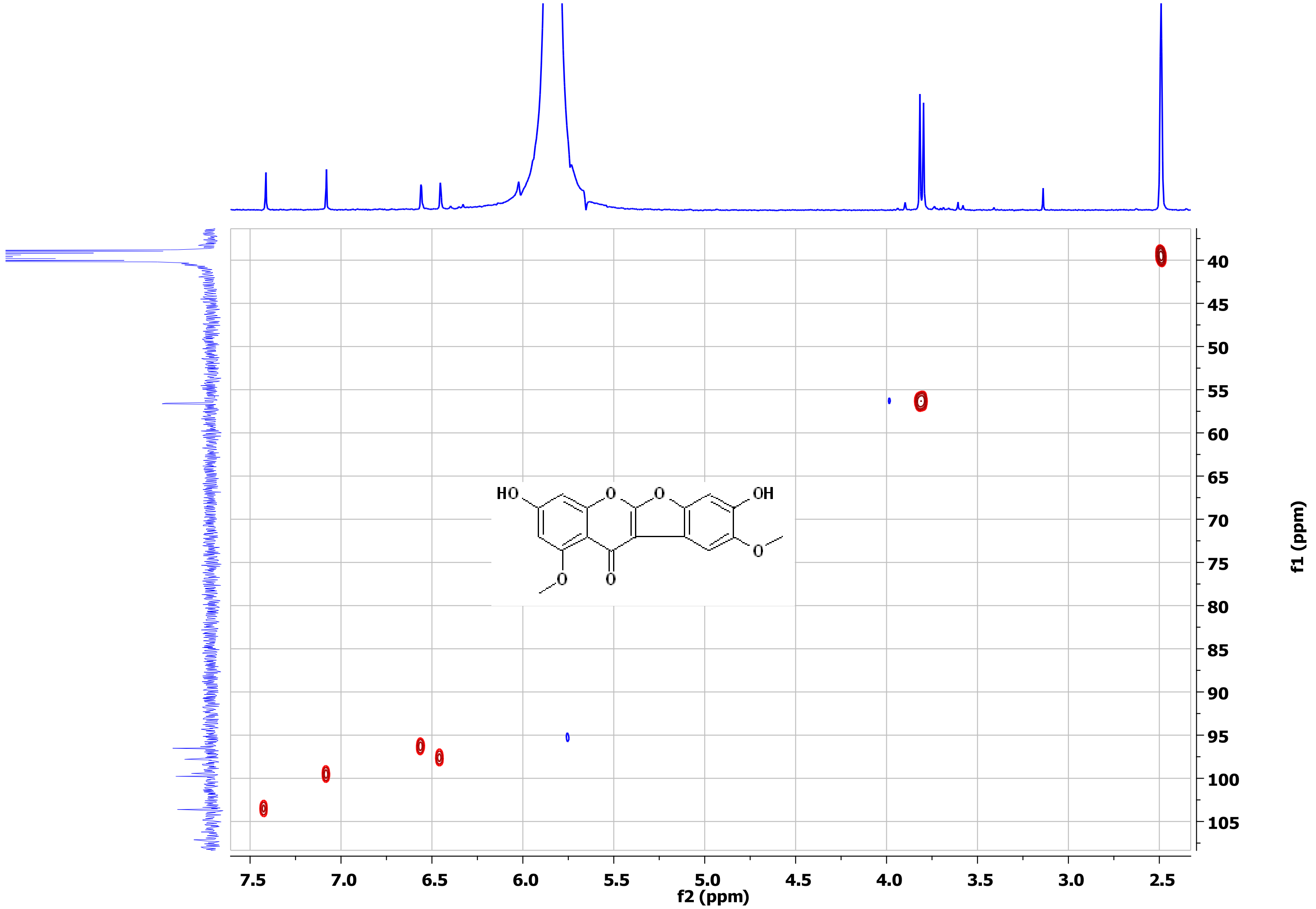

S18. Chiral HPLC analysis of the enantiomeric purity of erythrinin F (**3**).

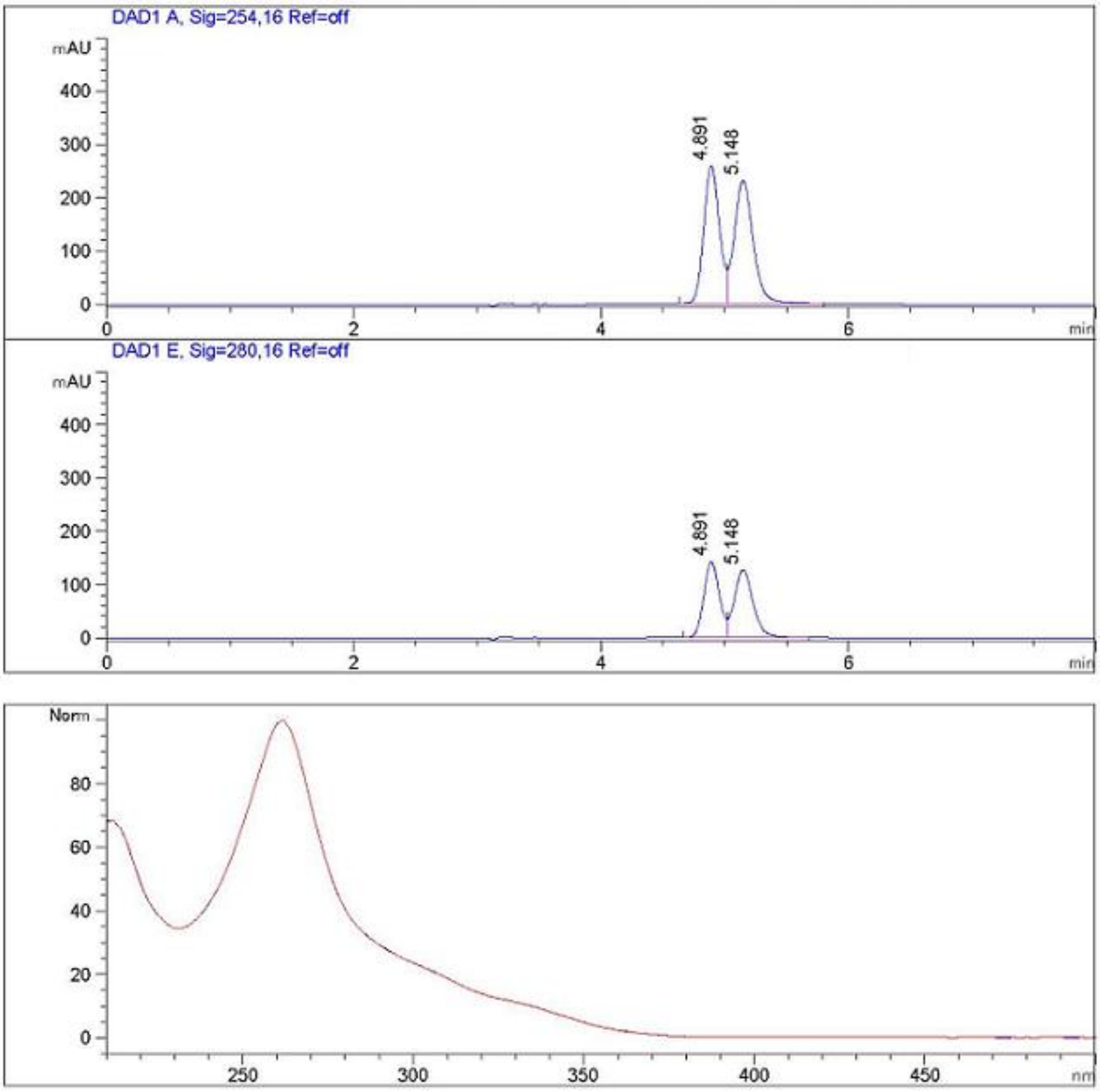

TCI Chiral MB-S column (5 μm, 4.6 × 250 mm)
(20% MeCN in H_2O over 10.0 min, 1.0 ml/min, 25°C)

S19. HPLC analysis and its UV spectrum of erythrinin D (**1**).

Product Number: BBP01120

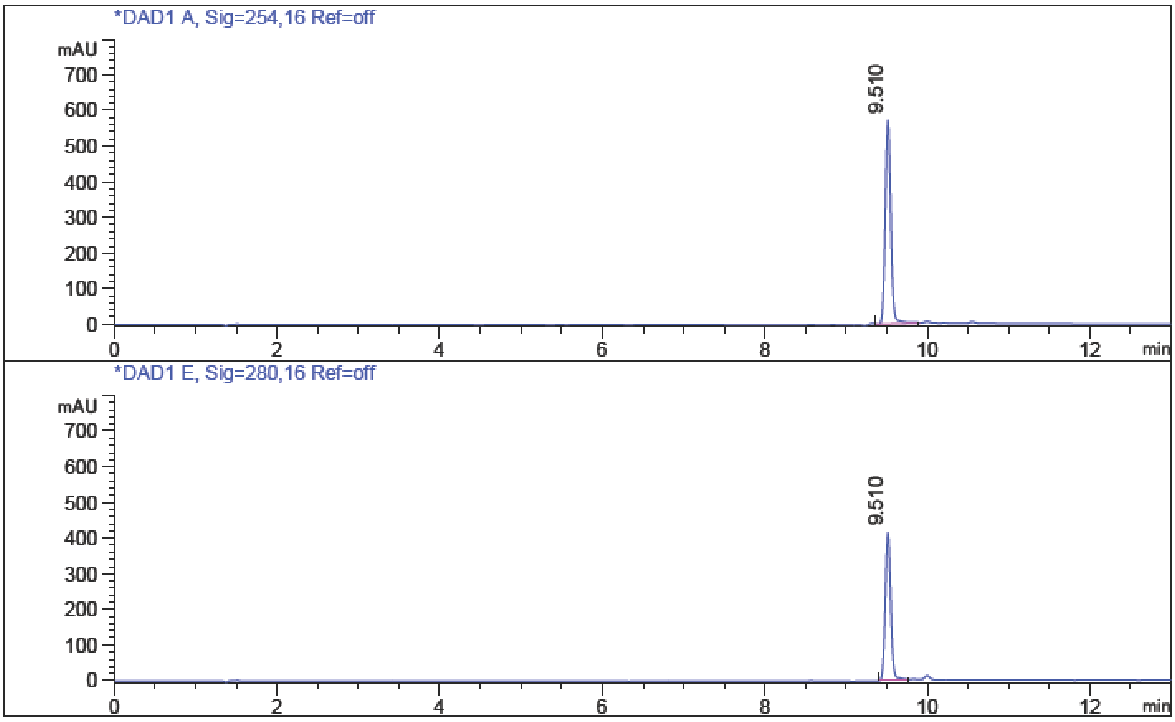

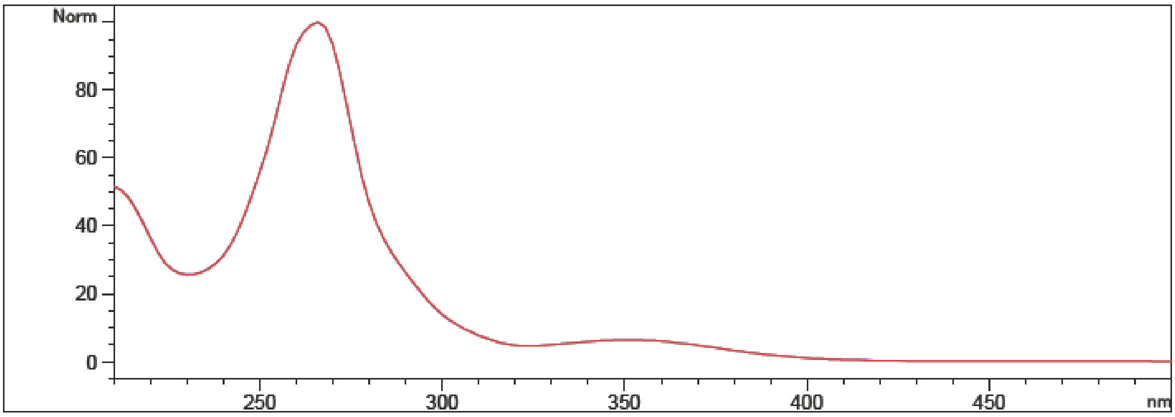

S20. HPLC analysis and its UV spectrum of erythrinin E (**2**).

Product Number: BBP01165

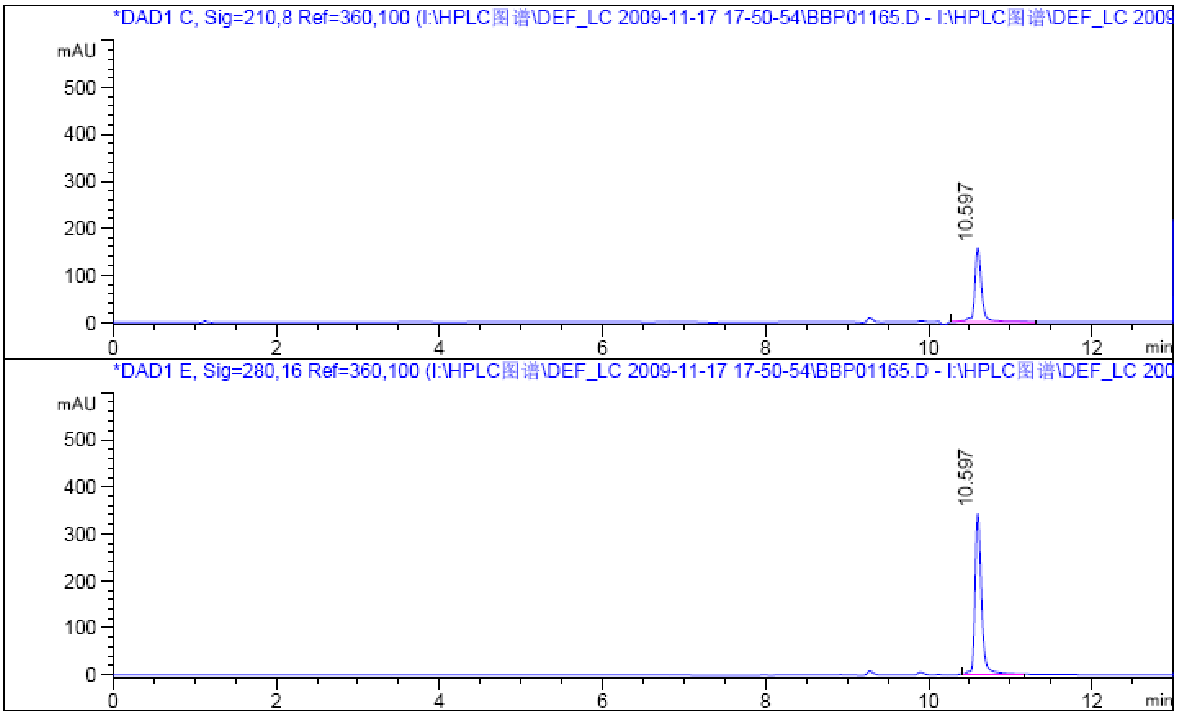

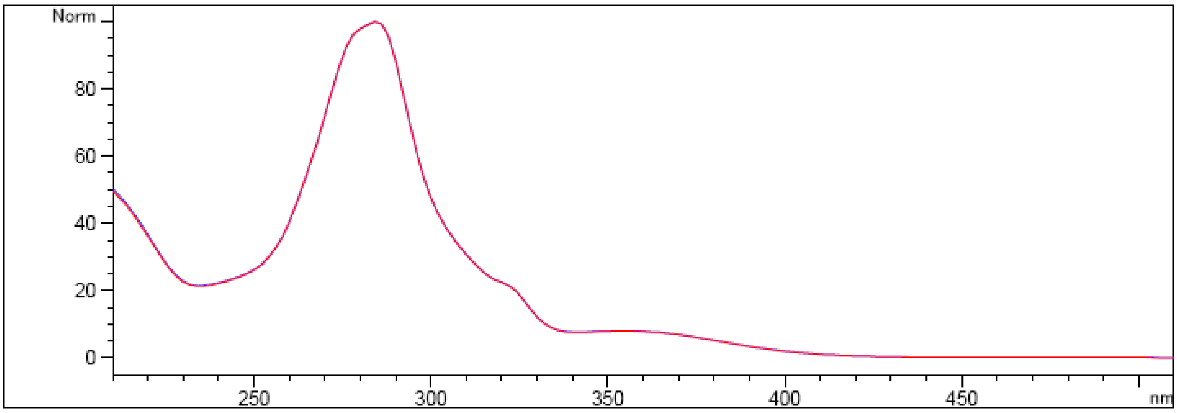

S21. HPLC analysis and its UV spectrum of erythrinin F (**3**).

Product Number: BBP02957

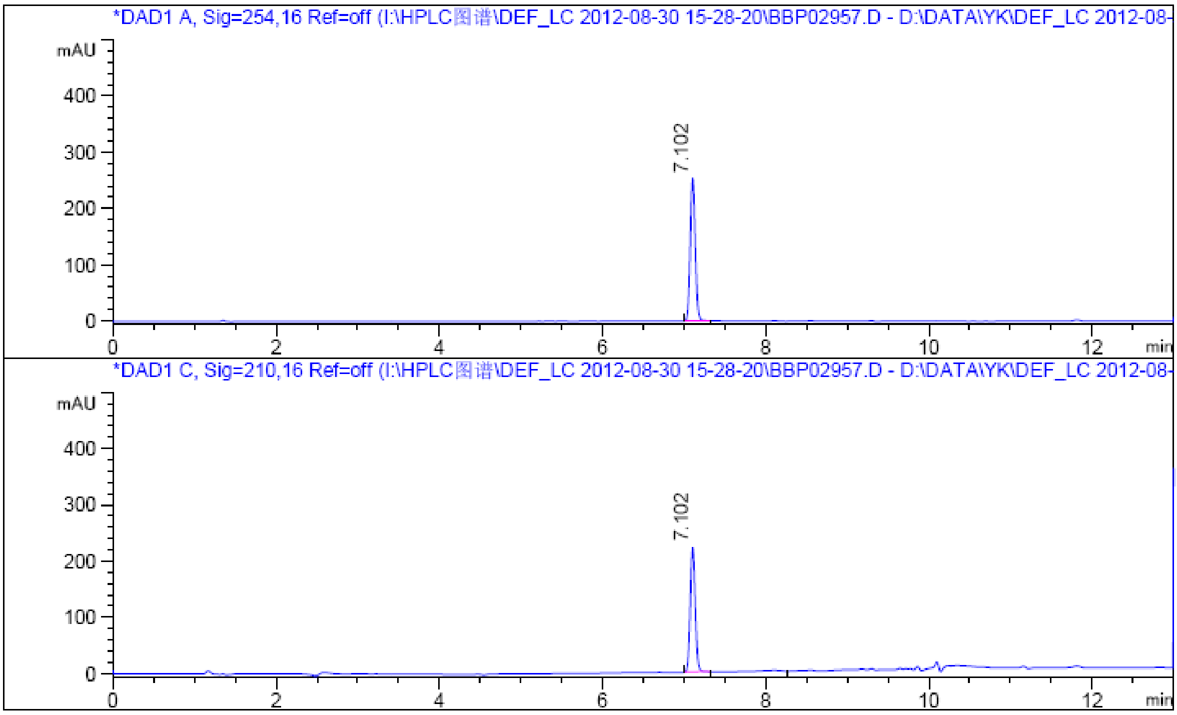

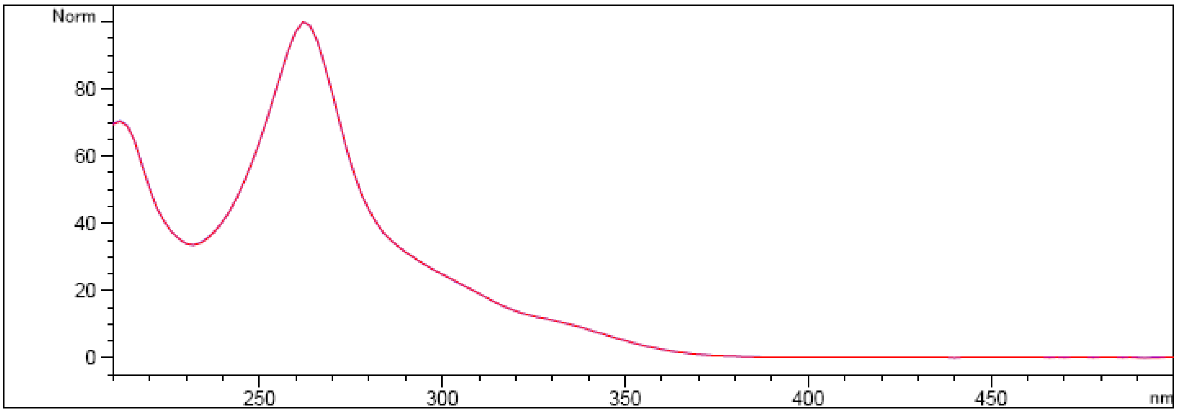

S22. HPLC analysis and its UV spectrum of erythrinin G (**4**).

Product Number: BBP02991

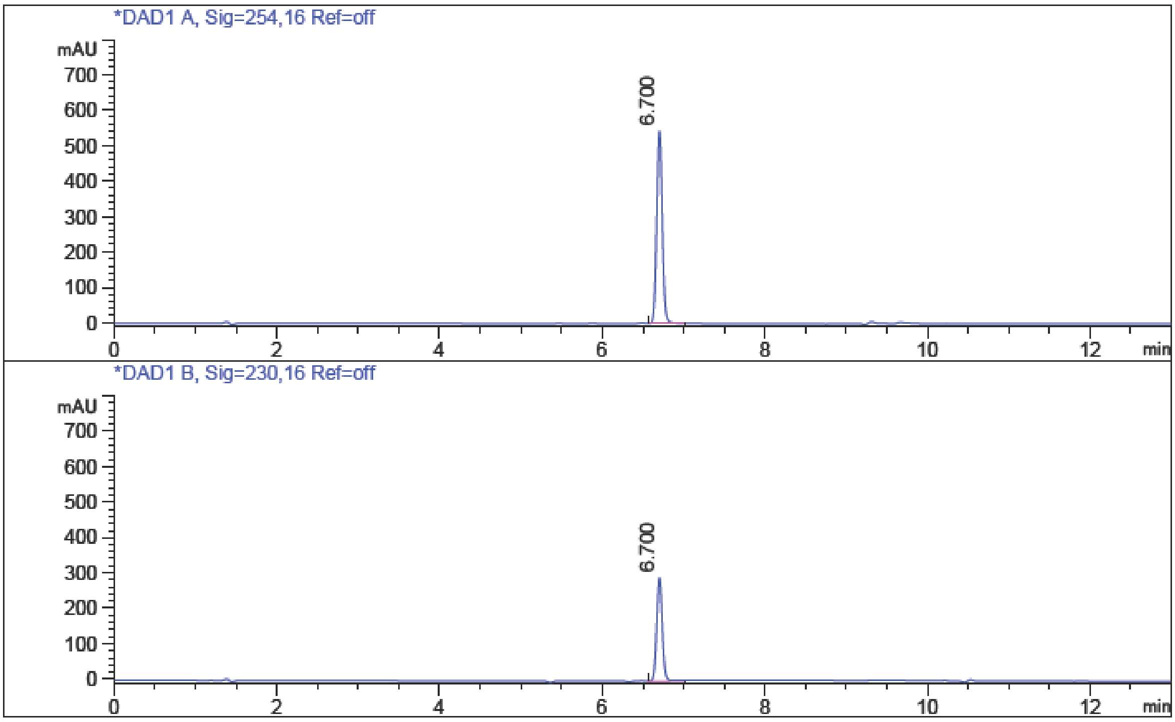

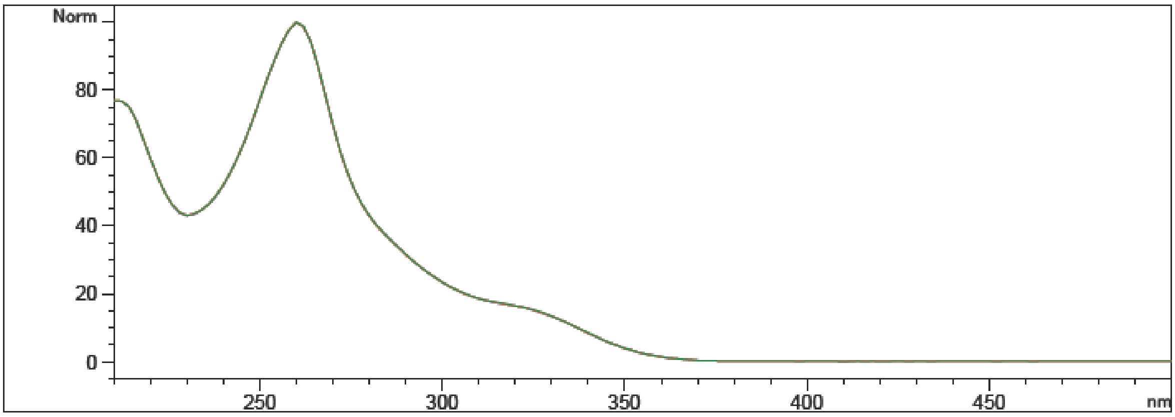

S23. HPLC analysis and its UV spectrum of erythrinin H (**5**).

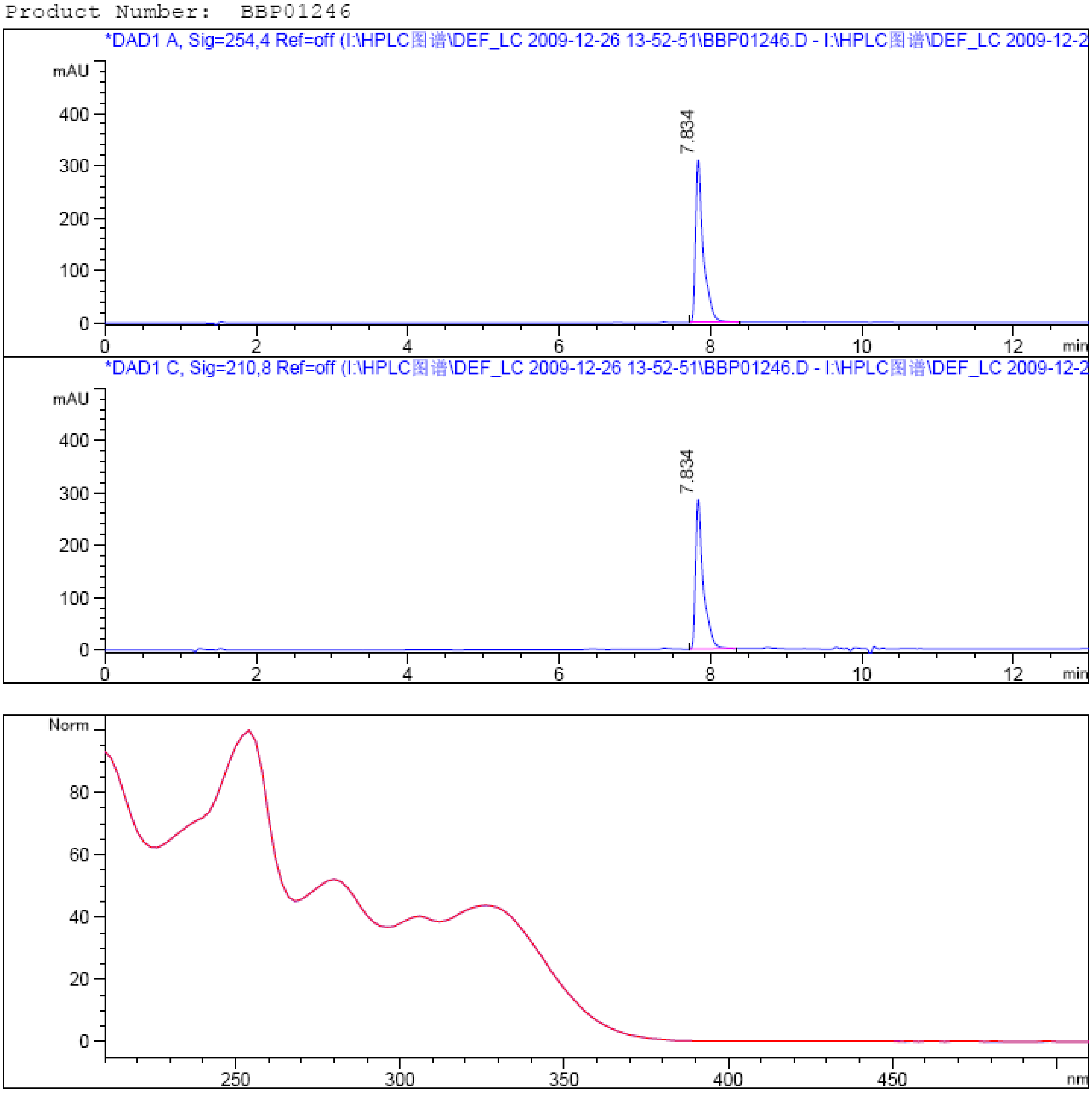

S24. HPLC analysis and its UV spectrum of 1″-*O*-methylerythrinin F (**6**).

Product Number: BBP02793

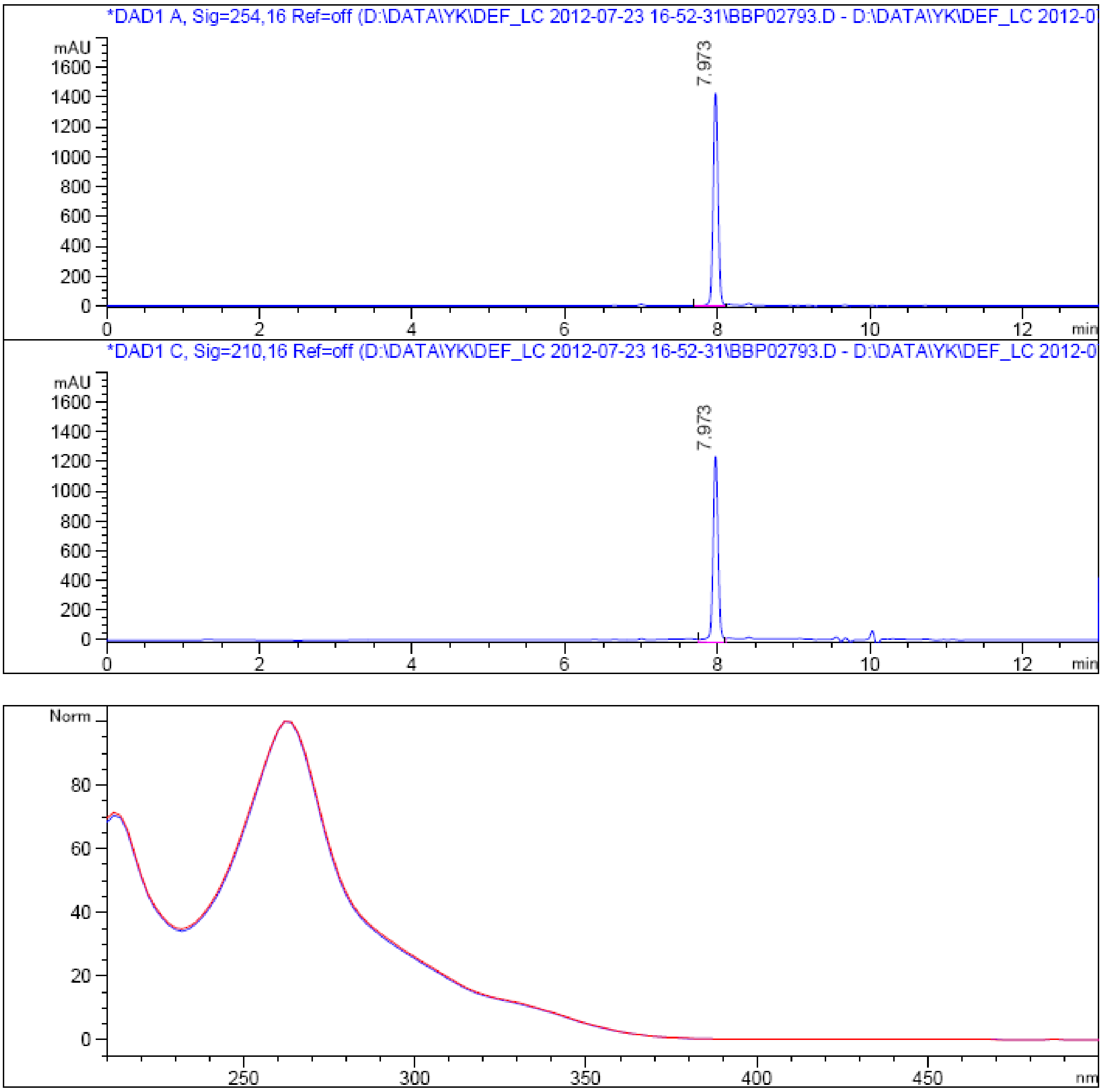

Supplement: Supplementary file 1 — Supplementary material, approximately 1.62 MB. [file 13659_2013_62_MOESM1_ESM.pdf]
